# Reverse‐engineering psychological resilience: A review and quantitative evaluation of psychometric instruments used in resilience research

**DOI:** 10.1111/aphw.70174

**Published:** 2026-07-01

**Authors:** Lukas G. Repnik, Jolana Wagner‐Skacel, Emanuel Jauk

**Affiliations:** ^1^ Department of Medical Psychology, Psychosomatics, and Psychotherapy Medical University of Graz Graz Austria; ^2^ Clinical Psychology and Psychotherapy Medical School Berlin Berlin Germany

**Keywords:** content analysis, evaluation, psychological resilience, psychometrics, questionnaires, review

## Abstract

Discussions on what psychological resilience is, its operationalizations, and approaches to measuring it have occupied the scientific community for decades. This manuscript (1) provides an overview of psychometric methods used to assess resilience, including key characteristics, and (2) evaluates resilience scales through a preregistered, content‐analytical evaluation of items based on expert ratings. Items from 83 resilience scales were empirically categorized using a literature‐based framework with three main categories (resilience as *a process*, *traits*, and *environmental factors*) and 12 corresponding subcategories. Across all scales, 31.79% of items reflected resilience as a process, 51.95% reflected five‐factor model traits, and 16.26% reflected environmental factors. An additional analysis weighted results according to citation frequency, showing how operationalizations influence the current research practice: From this perspective, 60.17% of the items relate to process‐related content, 34.59% to trait‐related content, and 5.24% to environment‐related content. This weighting reflects the prevalence and influence of specific scales in the field. The inter‐rater agreement across four raters was κ = .68. Overall, existing questionnaires predominantly cover trait‐based content, whereas widely used instruments tend to operationalize resilience as a dynamic process. We derive conceptually grounded recommendations for scale selection to improve clarity and comparability in future research.

## INTRODUCTION

### What resilience genuinely is and what it is not

Resilience is commonly defined as the capacity for positive adaptation in the presence of significant stressors or adversity (Masten, 2001). Although there is great diversity in the definition of psychological resilience, particularly across different disciplines, there is an overall consensus that both a substantial threat and the succeeding positive adaptation must be present in order to refer to resilience. It is generally assumed that psychological resilience is a phenomenon that arises from an individual's interaction with their environment (Rutter, 2006). The capacity for resilience is not understood as something that is inherent in humans by nature but is strengthened over a lifespan by successfully coping with problems and crises (Pangallo et al., 2015).

One problem in the field of resilience research is that the definitions sometimes already contain descriptions of key underlying factors of resilience. Therefore, a fundamental distinction needs to be made between the actual core of the resilience construct and its determinants. These are two utterly different issues. A comparison of the resilience questionnaires, their strategy for operationalizing resilience (observable on the basis of the identified resilience dimensions), and the definitions of resilience used highlights the issue that in fact two separated areas are intermingled. In order to make the diversity of resilience concepts visible not only narratively but also systematically, we summarize selected, frequently cited definitions in Table 1. Instead of providing as complete a historical overview as possible, we focus on definitions that are prototypical for different conceptual focal points: resilience as a personality trait, as the result of positive adaptation (outcome), or as a dynamic process (process). In addition, we consider a more recent meta‐model by Arnold et al. (2023), which explicitly integrates these three paradigms. The table classifies each definition according to the three‐level framework we use (trait, process, and environment), thus making it transparent how the theoretical logic of the resilience concepts is transferred to our subsequent content‐analytical coding of the questionnaire items. Although the definitions of resilience, such as those in Table 1, are useful, they do not capture the complex nature of resilience. Steven Southwick, for example, wrote in this regard “Determinants of resilience include a host of biological, psychological, social and cultural factors that interact with one another to determine how one responds to stressful experiences.” (Southwick et al., 2014, p. 2).

**TABLE 1:** Overview of influential definitions of psychological resilience and their conceptual positioning in the three‐level framework (trait, process, and environment).

Author/year	Brief description of the definition	Primary concept focus	Emphasized level(s)	Contribution to our framework
Wagnild and Young (1993, p. 165)	Resilience as a personality trait that buffers the effects of stress	Trait	Person/disposition	Prototypical trait definition; provides a reference point for our trait category (close to FFM).
Werner (1995, p. 81)	Resilience as “positive development” despite risk, maintenance of competence, recovery	Outcome/development	Person × environment (developmental process)	Establishes the idea of multiple outcome forms (positive level, competence under stress, recovery).
Luthar et al. (2000, p. 543)	Resilience as a dynamic process of positive adaptation under considerable stress	Process (dynamic, adaptive)	Process of adaptation	Canonical process definition; forms the conceptual basis for our process category.
Rutter (2006, p. 1)	Resilience as an interactive concept: resistance to or overcoming of risks	Process/interaction	Person–environment interaction	Emphasizes explicitly the interaction between person and environment; combines process and environment perspectives.
American Psychological Association, 2014, as cited in Southwick et al. (2014, p. 2)	Resilience as a process of “adapting well” and “bouncing back” after stress	Process (bouncing back)	Individual adaptation process	Frequently cited, practical process definition; anchors “bouncing back” in the framework.
Arnold et al. (2023)	Three paradigms of resilience research: trait, outcome, process; integrative model	Hybrid form/meta‐framework	Trait, process, outcome (meta‐level)	Legitimation of our three‐category framework; provides theoretical framework for item coding.

The fact that resilience depends upon a series of mutually interacting variables suggests that resilience itself is unlikely to reflect a dichotomous attribute that an individual may or may not exhibit. Rather, each individual is on a continuum between resilience and vulnerability (Zolkoski & Bullock, 2012).

One central question for resilience research therefore concerns the operationalization of the construct. There are clear differences when it comes to psychometric questionnaires and models that aim to measure or explain resilience. As a result, different focal points have been researched and used in parallel in the past. This has already been criticized due to the fact that the resilience construct is becoming increasingly blurred and the validity of psychometric procedures is affected (Luthar & Brown, 2007; Luthar & Cicchetti, 2000). In the past, several researchers have spoken out in favor of a critical evaluation, a standardized definition and operationalization (Cicchetti et al., 1993; Kumpfer, 1999; Luthar et al., 2000).

Resilience research has a long tradition within psychology and related disciplines. As early as the 1950s, the first longitudinal studies of “development under risk” were conducted and particularly through the work of Emmy E. Werner and Ruth S. Smith associated with the term resilience (Werner, 1993). Today, the findings of this research group are considered to be the cornerstone of resilience research. In particular, the aspects that lead to positive development despite the presence of risk conditions and the central influence of social support were well‐elaborated in this study and set the tone for subsequent projects and resilience models. Different research traditions have explored resilience from various angles, using qualitative interviews or case analyses, and eventually the development of psychometric questionnaires—to make resilience quantifiable and thus measurable. An economical and above all reliable assessment approach to measure resilience is not only interesting for research purposes but also has high clinical relevance as a predictor of recovery (Durish et al., 2019; Pan & Sánchez, 2022; Southwick et al., 2014). With the flourishing of positive psychology in the 1990s, the research interest around resilience was once again tremendously strengthened in the scientific community. Today, an almost overwhelming number of questionnaires exist to assess resilience. These range from general scales to domain‐specific questionnaires that look at resilience in different stages of life or in different contexts (family, education, work, etc.).

### Current perspectives on operationalization in resilience research

To date, 17 articles have reviewed psychometric instruments for measuring resilience (see methods for details on literature search). These reviews have dealt with different focal points of the resilience questionnaires. First of all, a series of validity assessments were carried out to operationalize resilience, which also recommend different questionnaires for different demands by considering the psychometric properties of the scales (Fisher & Law, 2021; Pangallo et al., 2015; Salisu & Hashim, 2017; Smith‐Osborne & Whitehill Bolton, 2013; Wadi et al., 2020; Windle et al., 2011).

Some of these review articles also make specific recommendations on the use of certain questionnaires. However, it can be seen that only a few questionnaires are mentioned in these recommendations, but they are mentioned frequently. These include different versions of the Resilience Scale (RS/‐5/‐11) (Wagnild & Young, 1993), different versions of the Connor Davidson Resilience Scale (CD‐RISC‐2/‐10/‐25) (Connor & Davidson, 2003), the Brief Resilient Coping Scale (BRCS) (Sinclair & Wallston, 2004), the Brief Resilience Scale (BRS) (Smith et al., 2008), and the Resilience Scale for Adults (RSA) (Friborg et al., 2003). At the same time, these questionnaires were also the most frequently cited in scientific publications, as can be seen from the overview in this article in Supporting Information S1 (Supporting Information S1 is hosted on the Open Science Framework [OSF] repository due to format restrictions and can be accessed at https://osf.io/6b7dg/).

However, existing reviews primarily examine those questionnaires that are frequently used in resilience research anyway. The aim of this review and quantitative evaluation is to provide a comprehensive overview of currently available measures of resilience, as well as an edited presentation of these scales so that research‐relevant information can be derived from them. In addition to the overview of resilience questionnaires, an evaluation of the resilience questionnaires was also conducted in this project, which includes a classification in a resilience framework designed within our project. Thereby, our study represents an ecological assessment of existing resilience questionnaires.

### The problem of operationalization in resilience questionnaires

Dominant concepts that can be derived from different research traditions are the definition of resilience as a capacity, an outcome, and a process (Arnold et al., 2023). The *trait and capacity approach* defines resilience as a more or less stable (personality) trait (Fredrickson et al., 2003; Hartmann et al., 2020; Shin et al., 2012). With the nomological network of psychological resilience as a trait further includes curiosity, sociability, optimism, conscientiousness, and a positive, future‐oriented attitude (Skodol, 2010).

The relationship between resilience and the personality dimensions of the five‐factor model is a topic that has already been well researched. For example, a meta‐analysis by Oshio et al. (2018) shows that the five personality dimensions correlate to varying degrees with psychological resilience. The authors report moderate average correlations for openness (*r* = 0.34), conscientiousness (*r* = 0.42), extraversion (*r* = 0.42), agreeableness (*r* = 0.31), and neuroticism (*r* = −0.46). The association between resilience and the five‐factor model of personality was also investigated by Waaktaar and Torgersen (2010). They presented a sample of adolescents with resilience scales, all of which defined resilience as a personality trait. The authors concluded that these resilience scales were not significantly stronger predictors of the ability to adapt to adversity than the Big Five personality traits.

What is generally reflected in the topic of resilience and accordingly also in the questionnaires on this construct are the considerable differences in definition. This variance is also reflected in the corresponding questionnaires. When examining the psychometric resilience questionnaires, it quickly becomes apparent that the focus of the content differs considerably. Although the process level, for example, is a central factor in the American Psychological Association's definition of resilience (American Psychological Association, 2014, as cited in Southwick et al., 2014), the questionnaires often tend to focus on general resources, environmental factors, or personality traits—but the process level, in particular, is often missing.

Furthermore, the divergence between the definitions of resilience used and the operationalization of measuring resilience is striking. This becomes apparent on closer inspection of the resilience questionnaires (Linnenluecke, 2017). Factor analyses of the questionnaires show that the individual factors often reflect individual aspects that lead to resilience but do not necessarily cover the core definitional aspects of resilience. The following examples illustrate this circumstance. The CD‐RISC‐25 comprises the following factor dimensions: (1) personal competence, high standards, and tenacity; (2) trust in one's instincts, tolerance of negative affect, and strengthening effects of stress; (3) positive acceptance of change and secure relationships; (4) control; and (5) spiritual influences (Connor & Davidson, 2003). Wagnild and Young's Resilience Scale includes the dimensions (1) personal competence and (2) acceptance of self and life (Wagnild & Young, 1993). A detailed overview of the dimensions of the respective resilience questionnaires can be found in the table in Supporting Information S1 (see OSF project repository). These underlying factors certainly have a logical direct or indirect relationship with resilience but do not constitute resilience per se.

However, there are also questionnaires that have a unidimensional factor solution (Block & Kremen, 2011; Smith et al., 2008) or the short versions of the Connor‐Davidson Resilience Scale (CD‐RISC‐10/‐2). Although this does not contradict a specific definition of resilience, the following tendency is evident. In the unidimensionally structured resilience questionnaires, the individual items are very closely aligned with the definitional criteria: “I tend to bounce back quickly after hard times” and “I usually come through difficult times with little trouble” (Smith et al., 2008). In multidimensional scales, on the other hand, resilience tends to be operationalized via the presence of specific psychological abilities or resources, and thus, they tend to summarize aspects that lead to resilience: “I believe in my own abilities” and “I have some close friends/family members who really care about me” (Friborg et al., 2005). With this point, we would like to emphasize once again that the operationalization of resilience in the questionnaires is subject to considerable variation.

Finally, resilience questionnaires also frequently cover other aspects that are associated with personality trait characteristics of an individual. Specifically, there is a large overlap with trait dimensions that correspond closely to the five‐factor model of personality. Some questionnaires are based on an item pool, which could give the impression of being a modified form of the NEO personality inventory (McCrae & Costa, 2010). This also raises the question of whether these questionnaires actually do justice to the resilience construct. Or do aspects of resilience, vulnerability, and personality blur into one big, vague concept of psychological functioning? In any case, the discriminatory validity of these questionnaires should be critically assessed.

A key challenge of resilience research is that, despite surface‐level similarities between existing measurement instruments, there is no guarantee that these scales actually measure the same conceptual content. Different operationalizations of resilience lead to a heterogeneity of content that significantly limits the comparability and generalizability of research results. Correlative studies that compare resilience scales with one another therefore only provide limited information about the underlying constructs, as the content of the measured dimensions is not specified.

This is where the present study takes an innovative methodological approach: Using a systematic content analysis procedure based on theory‐led expert ratings, we explicitly identify which theoretical aspects of psychological resilience are actually represented by different scales. This procedure overcomes the methodological limitations of purely correlative approaches and offers a clearer operationalization by making concrete construct content visible. This study thus makes a decisive contribution to reducing conceptual ambiguities and methodological weaknesses in resilience research and at the same time provides practical, evidence‐based recommendations for the selection of suitable measurement instruments.

### Theoretical models of resilience

Although resilience research has been advanced with great efforts in recent decades, there are only a few theoretical models that have attempted to model resilience as a concept in itself. Theoretical descriptions and models have tended to take a meta‐level approach and are generally concerned with the influence of risk and protective factors on human behavior and experience or as models for stress processing (Masten & Powell, 2003). These models therefore also stem more from the perspective of explaining psychopathology and its sustaining and non‐sustaining factors. Among the few explicitly developed resilience models, Kumpfer's framework model occupies a central position, as it describes resilience as a multi‐stage adaptation process in the dynamics between the individual and their environment. In the following, we will use this model as an example and relate it to our three‐level framework (trait, process, and environment)

### Kumpfer's resilience framework

Kumpfer's resilience model describes resilience as a dynamic adaptation process characterized by the interaction between stressors, individual dispositions, and environmental conditions. Central to this is that (a) stressors or challenges disrupt homeostasis, (b) environmental contexts (e.g. family, peers, and institutions) provide risk and protective factors, and (c) internal resilience factors (cognitive, emotional, behavioral, physical, and spiritual resources) are mobilized in a person–environment transaction process to enable a more or less successful reintegration (Kumpfer, 1999). Compared to this multi‐stage process model, our three‐category framework (trait, process, and environment) represents a deliberately simplified, measurement‐oriented abstraction. We assign dispositional characteristics, which are described as internal resilience factors in Kumpfer's model, to the trait level; adaptive coping and adaptation processes correspond to the process level; and contextual resources and stresses (e.g. social support, family, and institutional conditions) are represented at the environment level. Thus, our approach does not compete with Kumpfer's model but rather translates its central levels into a reduced category system that can be reliably applied at the level of individual questionnaire items and makes the content focus of common resilience scales comparable.

### The necessity of an evaluation

The main aim of this paper is a review and quantitative evaluation of currently available resilience questionnaires. The results of this evaluation, with the description of the content distribution of the scales, enable a targeted selection based on different criteria of questionnaires for own research projects. In this context, the question of how resilience is operationalized in a particular questionnaire is of central importance.

Our research design further aims to provide a quantitative description of the content of the questionnaire items. In doing so, we aim at a descriptive characterization (by means of indicating the percentage frequency and distribution of the categories). The aim of the evaluation process using expert ratings is also to assess how much resilience is actually included in the resilience questionnaires and where other focal points or constructs related to resilience tend to be assessed.

The first part of this review is of descriptive nature and will provide an overview of psychometric methods used to assess psychological resilience. We will first present the questionnaire, also including key characteristics for use in research. This is achieved with a targeted search for review articles that deal with psychometric methods for measuring resilience and a subsequent literature search to identify and include further scales.

The second part of this review is of evaluative nature. For this purpose, we will perform a two‐stage evaluation process of these questionnaires—which provides for a content‐analytical evaluation of the items by means of expert ratings. In Stage 1 of the evaluation process, four raters assign the items to one of three categories derived from the literature (Southwick et al., 2014) which, in turn, are divided into several sub‐categories: (1) process level (1a resilience, 1b vulnerability, 1c posttraumatic growth, and 1d resistance); (2) personality/trait level (2a openness, 2b conscientiousness, 2c extraversion, 2d agreeableness, 2e neuroticism, and 2f other trait factors); and (3) environmental level (3a social factors/social support, 3b other, and non‐social factors). For the rating process, a framework containing the above‐mentioned dimensions has been created for the raters, which can be found in Figure S1.

The development of this framework was ultimately built on the taxonomies presented earlier in this section, which was based on the following rationale. In particular, we followed the perspectives described in the literature (Southwick et al., 2014). From this, the levels of process and personality/trait were synthesized for the framework. For the process category, which in itself already comprises the outcome category, further sub‐dimensions were defined. These were also derived from the literature and are based on the level of development or function at which a person finds themselves after overcoming adversity. If the person develops to a higher level, this corresponds to post‐traumatic growth. If the level corresponds to the level before the adversity, this corresponds to the resilience or resistance category. Finally, if the person is unable to recover from adversity, this corresponds to vulnerability (Arnold et al., 2023). Corresponding graphical representations can be found in the publication by Arnold et al. (2023). For the personality/trait category, the dimensions of the five‐factor model of personality (openness, conscientiousness, extraversion, agreeableness, and neuroticism) (McCrae & Costa, 2010) were subsequently selected as sub‐dimensions. The Five‐Factor Model is currently the most established and empirically validated taxonomy of personality traits, both within the nonclinical and clinical range, and has also been used as a more general framework for the dimensional description of psychopathological symptoms (Kotov et al., 2017). It thus provides a general reference system that extends beyond the resilience literature. In terms of discriminant validity, using the Five‐Factor Model as a reference system allows us to investigate whether certain items of resilience measures may be subsumed under the long‐lasting personality dimensions or whether they assess aspects which go beyond these dimensions. The third category selected for the framework was environmental factors. This decision was largely based on the results of the central resilience studies from developmental psychology (Werner, 1993), which were able to empirically prove social support and other resources as important resilience factors. This was also implemented in the operationalization of resilience in some questionnaires, with some items also being formulated specifically for this area, which made the environmental factors appear to be an appropriate category for the framework. This framework was to be used in the first rating stage and, finally, by adding the free categories, expanded so that it could be used for a holistic evaluation of resilience questionnaires.

## MATERIALS AND METHOD

This review includes 83 psychometric scales for assessing resilience. In addition, there are a total of 17 overview and review articles that already provide an overview of resilience questionnaires. A complete overview can be found in Tables S1 and S2.

The conceptualization of the present project was preregistered using the Preregistration for Quantitative Research in Psychology (PRP‐QUANT) Template (https://prereg-psych.org/) and published on the OSF online repository of this project. Besides the outline of the project, the preregistration includes a detailed data analysis plan, as well as the research objectives

In order to compile the resilience questionnaires for the evaluation, the following databases were searched: EBSCOHost (APA PsycInfo, APA PsycArticles, PSYNDEX Literature with PSYNDEX Tests, MEDLINE, ERIC, and eBook Collection [EBSCOhost]). Searching PubMed and Google Scholar using the same search parameters resulted in duplicates. The following search parameters were included: (resilien* = TI) AND (questionnaire OR assess* OR scale* OR instrument OR measure* = TI). Results were restricted to English. The criterion for the inclusion of a questionnaire was that it was clear from the title of the instrument or from the description (or manual) that the questionnaire aims to assess resilience.

At the same time, we used a second search strategy to find further questionnaires. For this purpose, we specifically searched for previously published review articles that examined psychometric instruments for measuring resilience. For this purpose, we again searched the above‐mentioned scientific databases and expanded the search parameters by the following: review = TI. The literature search was conducted between January and May 2023.

In addition to the overview of questionnaires for measuring psychological resilience, the evaluation of these scales was a central goal of this project. For this part, it was necessary to obtain the respective questionnaires for the evaluation. The majority of the questionnaires were freely available and did not require licensing. For those not freely available, we asked the authors for permission. However, not all authors replied, and we were therefore unable to obtain all the questionnaires that had been found in the previous search. Finally, part of the scales required a license and was purchased for the evaluation.

Based on this search strategy, a total of 95 psychometric resilience scales were initially identified, 83 of which could be obtained for the rating and analyses. In Table S1, all references of the retrieved resilience scales are presented, as well as information on the reasons for exclusion (was not published in the original article, no response from authors, etc.) for those questionnaires that were not included in the evaluation (Table S2). Finally, Supporting Information S2 also includes a reference list for all the retrieved review articles on resilience scales.

Furthermore, Supporting Information S1 (hosted on OSF) contains detailed information on relevant aspects of the included resilience scale for this analysis. This includes information on how the scale was retrieved (from publication or directly from the author[s]); license type (open source vs. licensed); the number of citations on Google Scholar; author(s); year of publication; domain (general or specific); number of items; number and type of resilience dimension(s); the scale's definition of resilience; and scaling and rating format (self‐rating or external assessment). Supporting Information S1 also includes information on which psychometric instruments are discussed or mentioned in one of the review articles.

### Rating procedure

**FIGURE 1:**
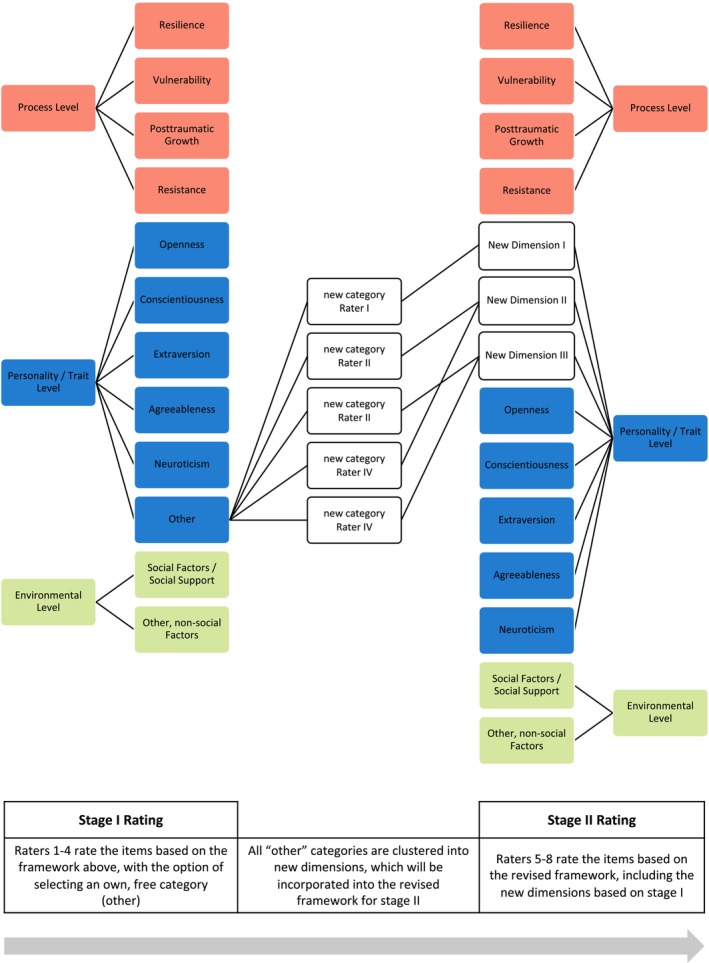
Flowchart of the rating procedure as planned in the preregistration.

For the rating process, experts from the fields of (clinical) psychology, psychiatry, and/or psychotherapy were selected. All raters possess at least a Master's degree in their respective field, with training in clinical and psychological assessment. A considerable factor in this endeavor is the number of raters used for the evaluation. We follow the example of other, comparable projects and define four raters per rating stage for the evaluation process. The whole rating process is thereby divided into two stages as can be seen in Figure 1.

In Stage 1, Raters 1–4 are presented with the list of resilience items and the framework derived from literature (see Figure 1, Stage 1 rating). For each item, raters should decide which level (process/trait/environment) and which dimension (process: resilience, vulnerability, posttraumatic growth or resistance; personality/trait: openness, conscientiousness, extraversion, agreeableness, neuroticism and other trait factors; environment: social factors/social support and other, non‐social factors) the item can best be assigned to. As can be seen from the framework (Figure 1/Figure S1), if an item cannot be clearly assigned to one of the dimensions, the raters have the option of assigning their own free category. However, we instructed raters to use this option as sparingly as possible. The additional categories can and should therefore also be used again if the item does not fit better into one of the categories from the framework.

After completion of Stage 1, the next step is to review the additional free categories that the raters suggested. This should ensure that the framework we provide for the rating actually covers the diversity of resilience items. The additional categories are collected and clustered in such a way that they can be integrated into the framework as an independent dimension. This revised version of the framework is then used as the basis for stage two of the rating process.

In Stage 2, all items are again presented to new raters. The experts are again asked to find the most appropriate category for each item. As already described, for the second rating stage, the categorization system used is based on the original framework and the additional categories suggested by the raters from stage one, although now without the possibility to select a free category individually. The new raters are again asked to assign all resilience items to the most appropriate category based on the updated framework.

The raters were instructed in detail about the study project and the rating procedure. All preparation material, including the rater instruction, can be found on the project's online repository on the OSF. The raters were thoroughly familiarized with the rating framework and the definitions of the individual subcategories. Before rating the questionnaire items, the raters underwent a joint rating session together with the PI, where the rating was practiced on freely made‐up sample items. Discrepancies were discussed until a common understanding could be agreed on. The raters should develop an understanding of the rating process and, in particular, be trained in the choice of a free category for raters of Stage 1.

Raters evaluate the items in a table in both the training data set and the main study. The rating files are structured in such a way that the rating is carried out by entering just one digit per item, and the selected category is filled in automatically to enable the raters to complete the rating as economically as possible. The experts rate the items blindly. This means they do not receive any additional information about the source or scale, so the individual items cannot be assigned to a resilience questionnaire. After successfully familiarizing themselves with the information material and the trial runs, the raters are ready to attempt the data set for the study. The order in which the items are presented in the study file is different for each rater in order to control for sequence effects (but the item order within each questionnaire is retained^1^).

Raters are asked to take regular breaks and, if their concentration is low, to continue the rating later. The raters also decide for themselves on the timing of their rating.

### Data analysis strategy

For the data analysis process, after the data set has been checked for missing values, the assignment of raters and their ratings is separated, and the raters are blinded. This ensures for the data analysis that an assignment of rater and rating is not possible. The assignment key was kept separate and secure. Data analysis involves calculating the inter‐rater agreement (between subjects) in addition to descriptive statistics (frequency distribution and percentage distribution).

Before aggregating and displaying the frequency distribution of the resilience questionnaires and items, inter‐rater reliability was calculated and checked. For this purpose, a modification of Cohen's kappa, namely, the kappa for multiple assessments (multi‐rater), was used. We aimed for at least a moderate level of agreement, which corresponds to a kappa value between .60 and .79, or in other words, 35–63% of the data should be reliable (McHugh, 2012). This was also defined in the preregistration, alongside some possibilities for further interventions if this goal is not achieved.

In addition, if we consider the number of citations of the respective resilience questionnaires, it becomes clear that not all psychometric instruments are cited with the same frequency and therefore have the same impact on research. Moreover, in the resilience research landscape, only a few resilience questionnaires are actually used as the measure of choice. In order to represent the actual state of the field of resilience research, we have also calculated the results of the ratings weighted according to the number of citations^2^ of the questionnaire on Google Scholar (as of July 2024). With an interesting shift in the result, the information on the number of citations can be found in Supporting Information S1 (hosted on OSF).

In order to provide a more comprehensive picture of the current state of resilience research, we analyzed the expert ratings from two complementary perspectives. The primary outcome of the study is the unweighted descriptive analysis of item distributions across all included scales, providing a direct overview of the theoretical content captured by each instrument. As a secondary outcome, the same analysis was weighted by the citation count of each questionnaire. This additional analytical step accounts for the differential impact of the scales in the scientific literature and offers insight into how widely used instruments shape the overall understanding of psychological resilience. By comparing these two perspectives—raw descriptives and citation‐weighted descriptives—we aimed to highlight potential disparities between theoretically grounded questionnaire content and the actual influence these instruments exert in empirical research practice.

To implement the citation‐weighted analysis, we applied a weighting procedure in which the individual contribution of each questionnaire to the overall dimensional distribution was adjusted according to its citation frequency in the scientific literature (Google Scholar, as of July 2024). Specifically, item‐level dimensional scores from the expert ratings were linked with citation counts of their respective questionnaires. Only instruments with available citation data were included in the weighted analysis. For each psychological dimension, a weighted mean was calculated using the number of citations as the weighting factor. This approach allows for a proportional integration of empirical impact, as frequently cited instruments exert greater influence on the weighted distribution. The resulting percentages provide an adjusted view of how resilience is operationalized in research practice, reflecting not only the theoretical structure of the instruments but also their practical relevance and dominance in the field. Aggregated values for higher order categories (process, trait, and environment) were computed by summing the corresponding weighted proportions.

The raw data from this project are published in the project's online repository on the OSF. The data set consists of the unique identification of the items (questionnaire and exact item number) and the corresponding ratings. For copyright reasons, original items cannot be published (this applies to the shared dataset as well as to the whole publication). The syntax and code for the data analysis can also be found on the repository.

For the data analysis, we used RStudio (Version 2024.12.0.467) running R (Version 4.4.1). The original data sets, alongside the R syntax, can be found online at the OSF project repository.

## RESULTS

The primary results of this study are descriptive in nature and show the content distribution of the questionnaires obtained during the rating process. In Stage 1, an initial inter‐rater agreement of the four raters could be achieved with a kappa coefficient of κ = .61. The strength of the agreement can be considered substantial (Landis & Koch, 1977; McHugh, 2012).

**FIGURE 2:**
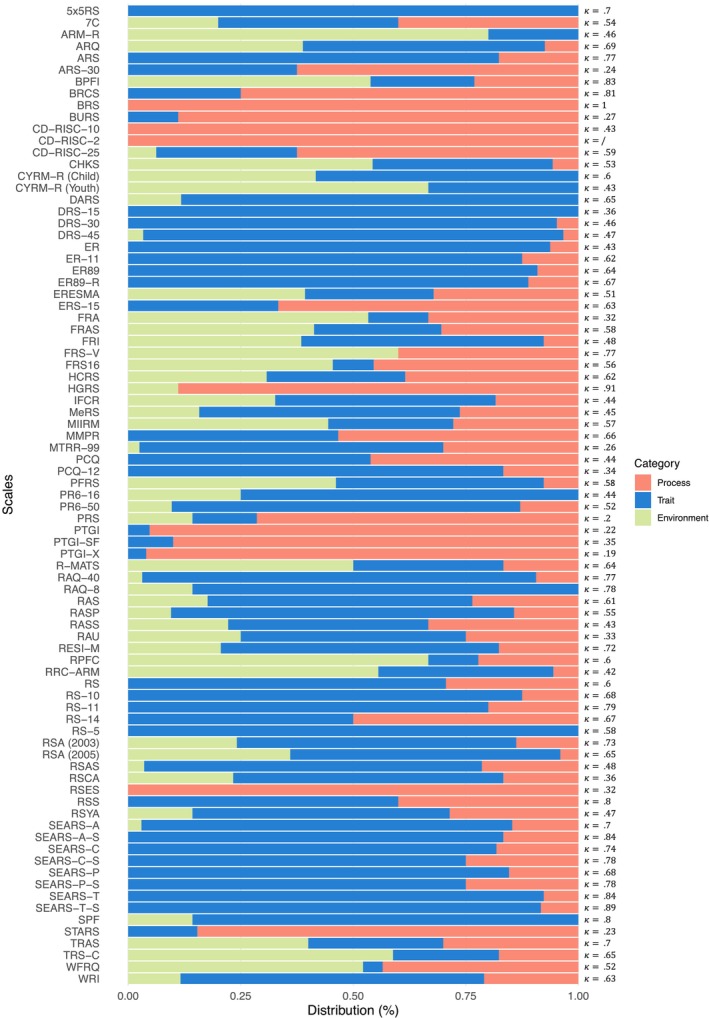
Inter‐rater reliability and distribution for the categories process, trait, and environment for each resilience questionnaire.

As stated in the preregistration, we examined whether the case‐by‐case exclusion of a single rater substantially influenced the overall rating in terms of the inter‐rater agreement. This was particularly the case with the exclusion of Rater 1, where the exclusion of this rater increased the kappa coefficient to κ = .71. Another finding at this point was that the four raters from the first stage only used the free category extremely rarely. Only two of the four raters used additional categories at all, and these only under 2% of all items. Rater 3 proposed the additional categories honesty (used in 0.18% of all items) and locus of control (used in 1.71% of all items). Rater 4 proposed the category purpose/meaning (used in 1.26% of all items). The fact that the use of additional categories was hardly necessary showed us that the proposed resilience framework is a good category scheme for assessing the items. These two findings were decisive for the further conduct of the study. Although the target inter‐rater agreement of κ > .60 was achieved in the first round of ratings, we additionally examined whether the exclusion of individual raters would lead to a further improvement in the homogeneity of the coding. A corresponding procedure was provided for in the pre‐registration in case the target agreement was not achieved; we used this logic here as a supplement in the sense of a robustness analysis. The analyses showed that the exclusion of Rater 1 in particular led to a clear increase in agreement. Because we also considered it methodologically essential to maintain complete ratings from four independent raters, we replaced Rater 1 with another expert with a comparable professional background and identical training. With this adjusted panel, we were able to achieve higher and more homogeneous inter‐rater agreement. This approach made it possible to achieve a more homogeneous assessment overall in this second rating phase. Therefore, we refer below to this rating constellation from the second phase for the further presentation of the results. As a result, the kappa coefficient could be increased to κ = .68 for Raters 2–5. The main results from the evaluation, that is, the percentage distribution of which categories were used by the raters to assess the items, can be found in Table 2. This is followed by another main result, which provides a more detailed view of the findings. These are shown in Figure 2. A presentation of the raters' assessment of the key content areas (levels) per questionnaire in the rating is shown, including the kappa coefficient of the inter‐rater agreement. Only items for which there was agreement in the rating were included in the presentation. Agreement is defined in this case as at least three raters having suggested the same dimension for an item. A more detailed representation of the distribution at the subdimension level can be found in Figure S2. An additional example that includes the level of disagreement is shown in Figure S3. Finally, the exact numerical distribution of the visualizations in Figure S3 can be found in Table S3.

**TABLE 2:** Percentage distribution for each category by rater; total with Rater 1 excluded.

	Total	Rater 1	Rater 2	Rater 3	Rater 4	Rater 5
Process level	26.68%	26.52%	22.38%	30.8%	25.66%	27.87%
Resilience	17.9%	7.2%	17.78%	20.22%	19.23%	14.36%
Vulnerability	0.54%	1.98%	0.09%	0.41%	0.14%	1.53%
Posttraumatic growth	4.75%	8.1%	3.92%	4.5%	4.59%	5.99%
Resistance	3.49%	9.23%	0.59%	5.67%	1.71%	5.99%
Personality/trait level	55.12%	45.61%	59.93%	53.58%	58.53%	48.45%
Openness	8.58%	7.25%	8.6%	8.6%	9.73%	7.38%
Conscientiousness	14.52%	13.73%	18.01%	11.44%	14.23%	14.41%
Extraversion	10.13%	8.24%	12.83%	8.73%	10.4%	8.55%
Agreeableness	10.55%	7.11%	10.72%	11.48%	11.26%	8.73%
Neuroticism	10.56%	9.28%	9.77%	11.44%	11.66%	9.73%
Other	1.58%	/	/	2.3%	2.7%	/
Environmental level	18.2%	27.87%	17.69%	15.62%	15.8%	23.68%
Social factors	14.46%	18.96%	14.45%	13.33%	13.1%	16.97%
Non‐social factors	3.74%	8.91%	3.24%	2.3%	2.7%	6.71%

### Results weighted by citation frequency

Results so far showed that the large number of resilience questionnaires provided broad coverage of the categories of process, (personality) trait and environmental factors, with a particular focus on the personality/trait level. But what about the representation of the questionnaires within research?

**FIGURE 3:**
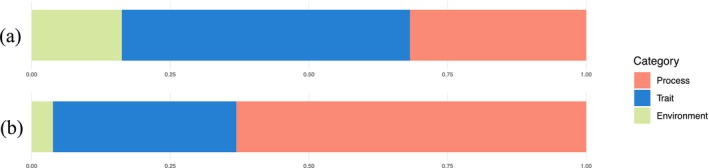
Mean distribution (a) and weighted mean distribution by citation (b) for the main categories process, trait, and environment, Agreement Raters 2–5.

The change in the results due to the added weighting can be summarized as follows: The levels of personality/trait and environment‐related items decreased, which was also evident in all sub‐dimensions (see Table 3). The number of process‐level items, however, strongly increased (+28.38%), which can be attributed essentially to the two sub‐dimensions resilience (+12.98%) and posttraumatic growth (+17.7). An overview of the mean distribution, weighted mean distribution, and their respective differences can be seen in Table 3 and Figure 3. These outcomes are determined on the basis of those items that were rated by at least three raters in the same category (meets the criterion for agreement).

**TABLE 3:** Mean distribution (%, *SD*, 95% CI range), citation‐weighted mean distribution (%, *SD*, 95% CI range), and Δ mean (weighted − unweighted) for all main categories and subdimensions (Raters 2–5).

	Unweighted distribution			Weighted distribution			ΔM
	*M*	*SD*	Range	*M*	*SD*	Range	
Process level	31.79%	26.43	18.21–29.58	60.17%	37.32	25.47–65.56	+28.38%
Resilience	23.06%	19.83	13.18–21.68	36.03%	31.53	11.14–46.9	+12.98%
Vulnerability	0.04%	0.24	0–0.08	0.02%	0.19	0–0.06	−0.02%
Posttraumatic growth	7.43%	18.35	2.14–9.77	23.13%	36.33	2.4–44.84	+15.7%
Resistance	1.26%	2.67	0.41–1.53	0.99%	1.94	0.13–1.83	−0.28%
Personality/trait level	51.95%	26.67	32.78–44.37	34.59%	24.53	16.51–41.7	−17.36%
Openness	10.78%	13.34	5.31–10.99	10.38%	12.69	3.77–14.96	−0.4%
Conscientiousness	14.86%	10.73	8.83–13.38	9.21%	9.07	3.75–12.67	−5.65%
Extraversion	9.36%	8.4	5.28–8.88	6.13%	7.99	1.95–9.37	−3.23%
Agreeableness	9.45%	10.44	4.85–9.31	4.69%	4.54	1.41–5.8	−4.75%
Neuroticism	7.51%	7.33	4.1–7.27	4.19%	5.3	1.67–5.4	−3.33%
Environmental level	16.26%	15.32	8.91–15.52	5.24%	9.19	2.11–7.58	−11.02%
Social factors	14.22%	13.9	7.74–13.63	4.89%	8.48	1.9–7.14	−9.33%
Non‐social factors	2.04%	3.44	0.82–2.27	0.35%	1.69	0.08–0.69	−1.69%

The citation‐weighted analysis served to give greater weight to more frequently used resilience scales in the literature. Although this approach does not necessarily reflect the conceptual quality or theoretical appropriateness of the instruments, it does reflect their visibility, use, and historical influence in the field of research. From this perspective, there is a relative increase in process‐related content in the highly cited instruments, whereas the unweighted consideration of all items continues to indicate a dominance of trait‐based operationalizations.

### Robustness/sensitivity analyses

To test robustness, we repeated the item classification and distribution analyses with the original four‐rater panel, including Rater 1 (majority rule). The resulting distributions (unweighted and citation‐weighted) at the process/trait/environment level and at the subdimension level were very similar to the patterns reported in the main text (panel without rater 1); there were only minor deviations, with no changes to the central conclusions. Details can be found in Supporting Information S3 (Table S3.1 and Table S3.2).

In addition to the main evaluation (only items with majority consensus), we conducted a systematic analysis of the disagreed items. We specifically calculated items‐scale‐wise uncertainty, entropy, and confusion patterns. Across all scales, the median disagreement rate is 26.09% (IQR: 14.67–36.18%; range: 0–73.33%). The mean entropy is 0.38 (all items) and 1.02 (disagreed items only). The confusion analyses show that the most common confusion is between process ↔ trait (10.67%), followed by trait ↔ environment (6.88%) and process ↔ environment (1.38%). At the subdimension level, the highest off‐diagonal pairs occur in content‐plausible boundary areas (see the supporting information). These findings specify where ambiguities arise without assuming a “correct” category and at the same time provide scale‐wise reference values (proportion ± 95% CI, entropy). These parameters indicate that ambiguities do occur but are primarily concentrated in the borderline areas between process and trait ratings. The higher mean entropy of the disagreed‐only items indicates a broader distribution of ratings (greater conceptual heterogeneity) without distorting the overall pattern. The confusion percentages quantify systematic areas of confusion between categories; higher off‐diagonal proportions (here primarily process ↔ trait) show where the categorical selectivity is weakest from a technical perspective. A detailed description of the analysis procedure, the associated results, tables, and figures can be found in Supporting Information S4.

## DISCUSSION

The aim of this study was to make a significant contribution to clarifying a fundamental challenge in resilience research: the lack of transparency regarding which theoretical constructs are actually operationalized and measured by established resilience questionnaires. By using a theory‐based, content‐analytical approach, the items from an extensive selection of psychometric resilience scales were systematically categorized and explicitly analyzed with regard to their core theoretical content. This approach differs fundamentally from correlative studies, which can be limited by the lack of content specification of the underlying constructs. The present study therefore not only closes an existing methodological gap but also takes an important step towards the conceptual clarification and operationalization of psychological resilience, which may have a lasting influence on future research.

Across all raters and questionnaires, an average distribution of the items with agreement was found in the categories process (31.79%), personality/trait (51.95%), and environmental factors (16.26%). When results are weighted according to the number of citations of the respective scales, the distribution shifts to 60.17% process, 34.59% personality/trait, and 5.24% environmental factors. To classify inter‐rater reliability, it is important to emphasize that κ quantifies methodological agreement in the coding process but does not determine content distributions per se. In Stage 1, the overall κ with four raters was κ = .61. Removing Rater 1 increased κ to .71, indicating that a considerable portion of the disagreements were attributable to this individual rater. With the replacement rater, the final coding achieved κ = .68 (substantial). A key point in this regard is that the sensitivity analyses show that the content patterns (both at the P/T/E and sub‐dimension levels) remained stable despite this methodological improvement and that the primary statements remain unchanged. The exclusion of Rater 1 thus improved the measurement quality (higher reliability) without systematically shifting the content interpretation.

Further sensitivity analyses showed that ambiguities are moderate on a scale basis (median 26.09% disagreed; entropy 0.38 and 1.02 for disagreed items) and occur primarily at the process ↔ trait interface (10.67%), whereas trait ↔ environment (6.88%) and process ↔ environment (1.38%) are less common.

Our main results showed an interesting picture regarding the main dimensions (process, trait/personality, and environment): The majority of the items covered the trait/personality spectrum. The already described overlap with personality traits according to the five‐factor model (Oshio et al., 2018; Waaktaar & Torgersen, 2010) can therefore also be found in this study. In particular, the personality dimension conscientiousness appears to play a central role for trait‐based resilience assessments.

At the process level, we evaluated whether items would correspond to the four categories: resilience, vulnerability, posttraumatic growth, and resistance. Our expectation was that the resilience category would be very dominant in the course of the rating. We found that within the process level, that our expectation was met and that the similar constructs (resistance, vulnerability and posttraumatic growth) could be well distinguished from resilience, and the respective definitions were a clear guideline for the raters.

The third main category, which covered environmental factors, also showed an interesting perspective. Here, social aspects in the sense of social support were primarily depicted in the items. This also confirms to some extent that social support is a key resilience factor, as described in the literature (Werner, 1993). Other environmental factors such as financial resources, spirituality, and hobbies therefore play a less central role in the questionnaires.

However, a different picture emerges when the results of the distribution are weighted according to the number of citations. The distribution in the process category increased by 28.38%, with categories personality/trait and environment decreasing by 17.36% and 11.02%, respectively. This shift in the citation‐weighted results of the distribution can be explained by the fact that some questionnaires in particular are now very significant due to the high number of citations. These include the CD‐RISC‐25 (over 14,500 citations), BRS (over 6500 citations), CD‐RISC‐10 (over 3500 citations), and RSA (over 2000 citations), for which the resilience category was predominantly selected, in addition the PTGI (over 10,000 citations), for which the category posttraumatic growth was chosen almost exclusively. All of these dimensions increase the process level correspondingly. A few questionnaires such as the RS (over 7500 citations) and the ER89 (over 4000 citations) were primarily assessed at the personality/trait level. All other questionnaires have a citation count of <2000, with over half of the questionnaires having a citation count of <120.

As astounding as the initial result of the study may seem, with personality/trait as the dominant dimension across all questionnaires, the whole picture looks much more coherent when the results are weighted according to the number of citations. This should give an idea of what the distribution might look like in the actual research landscape and thus give more weight to those questionnaires that are used more in scientific practice. In this context, it was shown that the process level was the most represented, accounting for just over 60% of the distribution.

A key finding of the weighted analysis is that the resilience questionnaires that are most frequently used and cited in the scientific literature predominantly contain items that, according to our categorization, are assigned to the “process” dimension. This finding is positive insofar as the “process” category most accurately reflects the core theoretical definition of psychological resilience. It encompasses the interplay between a significant threat to individual development and the resulting successful adaptation. Despite the existing conceptual heterogeneity in the field, these instruments thus reflect the central characteristic of the concept of resilience as a dynamic process of adaptation to adverse life circumstances, at least in their empirical application.

A driving scale in this context was the posttraumatic growth inventory (Tedeschi & Calhoun, 1996). At this point, it should be noted once again that posttraumatic growth can be understood as an extension of the concept of resilience.

Our analyses show that it was possible to evaluate the key content areas objectively and reliably (κ>.60).

### Implications

Which implications do the results of this study have for scientific practice? Overall, our analyses show that the landscape of resilience diagnostics must be interpreted in two ways: Although the unweighted overview favors trait‐based content, the citation‐weighted perspective makes clear that process‐related operationalizations have a relatively strong influence on current research practice. Though this suggests that process‐based conceptions of vulnerability have impacted the field more strongly, we note that citation does not necessarily reflect conceptual validity or superiority. An informed choice of scale should therefore explicitly consider whether an instrument primarily captures dispositional characteristics, dynamic adaptation processes, or contextual resources—and to what extent this fits the respective research or application goal. The central message of this study is that the heterogeneity of psychological resilience definitions also reflects in scales for its assessment. Resilience questionnaires cover a broad variety of underlying dimensions that are used to operationalize resilience. This also comes with the downside that it is difficult to compare different findings on resilience, and the comparability of studies is often only possible with reservations. If studies using different questionnaires to assess resilience are compared with each other, there is a high risk of a jingle fallacy, that is, assuming that two constructs are similar, because they bear the same name (Kelley, 1927). For this reason, a comparison of studies that have used different resilience questionnaires is not always a valid approach. In our view, this procedure requires a careful assessment of the questionnaires in advance and a good rationale as to why the comparison is valid.

In addition, the results of this study show a large overlap with personality dimensions. The majority of the items in the resilience questionnaires could be assigned to the dimensions of the five‐factor model. This can be explained, among other things, by the fact that resilience was understood as a personality trait in early research (Wagnild & Young, 1993). It was only later that the focus in the definition of resilience was directed more towards the outcome or at a process level (Luthar et al., 2000). As described above, the relationship between psychological resilience and the five‐factor model is well researched (Foumani et al., 2015; Fushimi & Imori, 2015; Mansouri et al., 2015; Sarubin et al., 2015). This concerns associations with individual personality dimensions, as well as the predictive validity of resilience questionnaires (Oshio et al., 2018; Waaktaar & Torgersen, 2010). These findings suggest that it may be possible to create a resilience index on the basis of a personality assessment, for example, using a questionnaire on the five‐factor model dimensions, which can then be used as a marker for an individual's ability to recover.

### Discussion of specific scales and recommendations for research and practice

In this section, a handful of selected scales are presented in more detail. The selection of these scales is based on the factors of how specifically the scale depicts a certain level of the framework (process, trait, or environment), how high the inter‐rater reliability of the rating was, and how strongly the questionnaire is represented in terms of citation numbers. This should also provide a decision‐making aid for the selection of scales for scientific projects. Specific recommendations in this regard are summarized again at the end of this section.

For the *process* level, two questionnaires stood out in particular. The BRS (Smith et al., 2008) reached a perfect inter‐rater agreement of 1. Although the scale consists of only six items, these were consistently rated by all raters on the resilience dimension. With more than 6500 citations, the BRS is frequently used in scientific publications, especially because the assessment of resilience with this scale is particularly economical. In their initial study, Smith et al. (2008) reported a unidimensional factor composition in the BRS, which could explain 55–67% of the variance in four different samples tested with PCA. They reached a satisfactory internal consistency of α = .80–.91. Other validation studies reported lower internal consistency (α = .71) with a two dimensional factor structure in a sample of *n* = 511 Chinese university undergraduate students (Fung, 2020). Another validation study also reported a two‐factor structure for the BRS with an α = .80 in a sample of *n* = 2272 Greek adults of the general population (Kyriazos et al., 2018).

Another scale worth mentioning is the posttraumatic growth inventory (PTGI) (Tedeschi & Calhoun, 1996). The inter‐rater agreement of this scale was κ = .22. For these agreed items, the raters assigned 95.24% of the items to the dimension posttraumatic growth and 4.76% to the dimension openness. The low inter‐rater agreement of this scale could be explained by the fact that some of the PTGI factors described by the authors incorporate the definitional criteria of posttraumatic growth quite well (e.g. new possibilities and appreciation of life), whereas other factors (e.g. relating to others, personal strength, and spiritual change), on the other hand, might represent very specific resources. The authors reached an internal consistency of α = .94 in their original study (Tedeschi & Calhoun, 1996). Other validation studies could replicate the five factor structure of the PTGI with an internal consistency of α = .96 in a sample of *n* = 273 adult males who reported experiencing clergy‐perpetrated childhood sexual abuse (Williams et al., 2015). The PTGI also represents one of the most cited scales with over 10,000 citations today.

For the *personality/trait* level, the Resilience Scale (RS) (Wagnild & Young, 1993) was particularly significant. The RS is one of the first scales that soon achieved a major academic application and has been cited 7575 times since then. The RS achieved an inter‐rater reliability of κ = .60 and primarily covered dimensions of the personality/trait level (48%): openness (12%), conscientiousness (20%), extraversion (8%), agreeableness (4%), neuroticism (4%), and 20% process: resilience (16%) and resistance (4%). The result with the emphasis on the trait level also reflects the authors' definitional criteria: “[Resilience is] a personality characteristic that moderates the negative effects of stress and promotes adaptation.” (Wagnild & Young, 1993, p. 165). The authors could identify a two‐factor structure for the RS using CFA: (1) personal competence and (2) acceptance of self and life. The authors reached an internal consistency of α = .91 in their original study, which could be replicated by other validation studies in older adults (*n* = 599) and α = .94 (Leppert et al., 2005) or Finnish participants (*n* = 243) with α = .90 (Losoi et al., 2013).

For the *environmental* level, Baruth Protective Factor's Inventory (BPFI) (Baruth & Caroll, 2002) turned out to be prominent for this category. The scale has been cited over 250 times and reached an inter‐rater agreement of κ = .83 in this project. The raters assigned the items of the BPFI with the following distribution: resilience (20%), openness (6.66%), conscientiousness (6.66%), extraversion (6.66%), social factors (33.33%), and other, non‐social factors (13.33%). Four factors (adaptable personality, supportive environment, fewer stressors, and compensating experiences) are described by the authors. A validation study in *n* = 200 nursing students described three factors for the BPFI with an α=.66–.93 (Ali‐Abadi et al., 2020).

Over all, the Connor‐Davidson Resilience Scale (CD‐RISC‐25) (Connor & Davidson, 2003) and the Resilience Scale for Adults (RSA 2003) (Friborg et al., 2003) showed a sound distribution across all three main categories.

With over 14,500 citations, the CD‐RISC‐25 is the most frequently cited scale examined in this study. The CD‐RISC‐25 reached an inter‐rater agreement of κ = .59 and a distribution of the dimension as follows: resilience (36%), posttraumatic growth (4%), openness (4%), conscientiousness (4%), agreeableness (8%), neuroticism (4%), and social factors/social support (4%).

The authors described a five‐factor composition for the CD‐RISC‐25: (1) personal competence, high standards, and tenacity; (2) trust in one's instincts, tolerance of negative affect, and strengthening effects of stress; (3) positive acceptance of change, and secure relationships; (4) control; and (5) spiritual influences. The authors reported an internal consistency of α = 0.89.

Other validation studies could not replicate the five‐factor solution of the CD‐RISC‐25. They reported two factors (α = 0.90–0.91; Green et al., 2014), three factors (α = 0.91; Yu & Zhang, 2007), or four factors (α = 0.75; Wu et al., 2017).

Finally, the RSA is another prominent scale with over 2000 citations. The scale reached an inter‐rater agreement of κ = .73 within our analyses. The rating of the items showed the following distribution: resilience (11.11%), conscientiousness (16.67%), extraversion (25%), agreeableness (5.56%), neuroticism (2.78%), and social factors/social support (19.45%). The authors described a five‐factor composition for the scale (1) personal competence, (2) social competence, (3) family coherence, (4) social support, and (5) personal structure with α = 0.67–0.90. A validation study found a six‐factor solution in a community sample of *n* = 805 adults with Hispanic Latin‐American background, with α = 0.90 (Morote et al., 2017).

The processual representation of resilience is covered in particular by the BRS (Smith et al., 2008) and PTGI (Tedeschi & Calhoun, 1996). The BRS had an excellent rating result in this study and can be fully assigned to the resilience category. With six items, the BRS is also a very economical measure. The PTGI also has the advantage that it is very well established as a measure in the scientific field. This scale should be used in particular when research interests focus on the specific factor of personal development as a result of a crisis. Posttraumatic growth goes beyond mere recovery from an adverse event and presupposes that personal development and personal growth have occurred as a result of overcoming a stressor.

A particularly suited choice for a personality‐ or trait‐based assessment of resilience is definitely the RS (Wagnild & Young, 1993). This scale, which is excellently established in science, covers this trait approach ideally.

If resilience shall be measured via environmental factors, social support, and other resources, we recommend choosing the BPFI (Baruth & Caroll, 2002). It covers these dimensions well and is a good choice for this purpose.

The CD‐RISC‐25 (Connor & Davidson, 2003) is certainly the scale of choice for use in a rather broader range of content. However, as a licensed instrument, there is a charge for using it for research purposes. A good alternative at this point seems to be the RSA (Friborg et al., 2003), providing a good combination of the three‐level process, trait, and environment.

In addition to the recommendations based on our resilience framework, we provide context‐specific guidance on selecting resilience scales. The key implication is that the label “resilience” covers different construct domains: process‐oriented adaptation (“bouncing back”; e.g. BRS), post‐traumatic growth (PTGI), trait‐related resources (RS, parts of CD‐RISC‐25, RSA), and environmental and contextual factors (BPFI, social subscales of RSA). For clinical practice, intervention developers, and policymakers, this means that resilience should not be measured “in general” but rather that the focus of the respective application should be clarified in advance. Process indicators are typically closer to current coping mechanisms and may show greater modification sensitivity (short‐ to medium‐term changeability under intervention). Trait‐related measures tend to reflect dispositions that, by definition, are relatively stable but may change over a longer time course. Environmental/contextual measures address structural resources and barriers (e.g. social support and organizational conditions) and are therefore particularly relevant for systemic interventions and health policies. Accordingly, instruments should be selected to suit the intended goal.

For clinical and intervention‐related questions that focus on short‐term changes in coping with stress, process‐focused scales such as the BRS are recommended. For those interested in qualitative developmental gains, the Posttraumatic Growth Inventory (PTGI) is also recommended. Development and personality research tends to benefit more from trait‐oriented instruments such as the RS and the personality‐related subscales of the Resilience Scale for Adults (RSA). For work and organizational psychology contexts and preventive health programs, on the other hand, instruments that map contextual protective factors and social resources (e.g. BPFI, social, and family RSA subscales) are useful. Multidimensional scales such as the CD‐RISC‐25 and the RSA should be used not only for overall values but also for their subdimensions: Process‐related factors may be used as primary target variables of an intervention, whereas trait‐ and context‐related factors can be used as initial conditions or moderators. Table 4 summarizes these scale‐ and context‐specific recommendations, providing users with practical support for informed decision‐making.

**TABLE 4:** Recommended resilience scales by context and construct focus.

Context/setting	Primary focus	Recommended scales (examples)	Instructions for use
Clinical/intervention (psychotherapy, rehabilitation)	Acute adaptation processes (“bouncing back”)	BRS (total score); process‐oriented subscales of the CD‐RISC‐25	For measuring progress and outcomes; sensitive to short‐term changes.
Clinical focus on growth after trauma	Post‐traumatic growth	PTGI (total score, subscales if applicable)	Use in addition to BRS/process measures when qualitative developmental gains are relevant.
Developmental/personality research	Trait‐based resilience resources	RS (total score); trait‐oriented subscales of the RSA	Rather stable dispositions; suitable for longitudinal and structural modeling studies.
Work and organizational psychology	Resources in the work and social context	RSA (social/family subscales), BPFI	Focus on social support, organizational resources, team and leadership context.
Public health/surveillance	Economic screening measures, basic indicators	BRS (short measure); BPFI (context/resource focus)	For large samples; combination of individual adaptability and structural protective factors.

A further development to consider in future resilience research involves the increasing use of ecologically valid and geographically informed assessment methods in natural environments. Recent studies using ecological momentary assessment (EMA) and geographically explicit EMA (GEMA) indicate that resilience‐related processes may be measured more accurately when individuals are assessed repeatedly in their daily environments and connected to contextual indicators such as green space exposure, ambient features, or place‐based stressors. From this perspective, traditional resilience scales might need two adjustments: first, by adding state‐sensitive items that evaluate situational recovery, regulation, and coping specifically related to certain environments and, second, by broadening environmental subdimensions beyond immediate social support to include contact with nature, restorative settings, and ecological resources. These changes would not replace existing trait‐ or process‐focused instruments but would complement them by increasing ecological validity and allowing for a more context‐aware assessment of resilience as it occurs in real‐world settings. This is especially relevant given recent theoretical work framing nature contact as a biopsychosocial resource for resilience and systematic reviews showing that geographically informed EMA studies consistently connect exposure to nature in daily life with improved mental health and well‐being (Christensen et al., 2025; Lohani & Blodgett, 2025; White et al., 2023).

### Limitations and future directions

Although this study offers valuable insights, it is essential to acknowledge certain limitations that may have impacted the findings and suggest potential avenues for future research. The study focused primarily on those items that were assigned to the same dimension by the raters with a certain level of agreement. Those items that did not achieve this level of agreement were not considered further in the study, which is certainly a limiting factor and leads to the loss of potentially significant data. Although it was possible to evaluate the item content in an objective and reliable manner at the overall level, there was still a wide range across the individual questionnaires. In some cases, perfect agreement was achieved, whereas other questionnaires only achieved an agreement of κ = .19 in the rating. This circumstance, or in particular those questionnaires that achieved a very low inter‐rater agreement, should possibly be checked again in terms of content.

The decision to exclude Rater 1 and replace them with Rater 5, though methodologically critical, was made due to specific findings during the initial rating phase. This procedure was specified in the pre‐registration as the following steps to be taken if (a) the target inter‐rater agreement of κ > .60 was not achieved or (b) one rater systematically skewed the overall result. Because the latter was the case in our analyses, but the three remaining raters had a very high level of agreement in their ratings, we decided to take this approach for the final analyses. Importantly, the target inter‐rater agreement (κ > .60) was achieved already in the first rating stage, with the initial four raters, indicating substantial agreement. However, Rater 1's influence significantly skewed the results downward, unlike other raters. Replacing rater 1 led to a more homogeneous and consistent overall rating, which we consider more meaningful. Rater 5 was selected with the same professional and educational background as Rater 1. This replacement was motivated purely by psychometric considerations, specifically to reduce the undue impact of any single deviant rater. All raters were kept blind to scale prominence and citation counts and used the same manual. Our sensitivity analyses (Supporting Information S3) show that the primary distributional patterns stay consistent whether or not Rater 1 was included, indicating no systematic bias from this choice. Contrary to expectations, the Stage 1 ratings showed little to no use of new categories, suggesting the suitability of the given classification framework.

A key consideration in the study's planning was determining the order in which items should be presented to the raters. Although complete randomization was considered, it was rejected due to high switching costs and the risk of inflating reliability by making patterns more recognizable to raters. Although some concerns arose about raters developing heuristics if items were blocked by subscale, in practice, most questionnaires already randomized their subscales. Thus, we retained the original order intended by the questionnaire authors. To address rater fatigue and maintain consistency, we divided the items into four blocks, presented in different sequences to each rater, while keeping the order within blocks unchanged.

We believe that with the proposed framework, a two‐stage rating procedure in which the categories collected in the first stage extend the framework for the second stage, we have developed a methodologically very sophisticated procedure. Future studies should pay more attention to the items that did not achieve sufficient agreement. These items could, for example, be used again in a joint discussion between the raters in order to find a consensus solution for all items. In this way, a complete and unambiguous rating of all items could be achieved. Future studies should also take a closer look at the defining criteria of resilience. In order to address the problem of different definitions, a consensus is first needed within the scientific community. The first steps and recommendations in this direction have already been taken. The Integrative Model of the Three Paradigms of Resilience (Arnold et al., 2023) is a particularly practical guide for classifying the resilience dimensions. This is the prerequisite for further steps at the level of psychometric assessment. At this point, specific recommendations regarding the selection of resilience questionnaires that cover the previously defined criteria for resilience in particular would be important in order to enable a valid operationalization of resilience.

Another important aspect concerns the cultural and contextual generalizability of our findings. The resilience questionnaires included in this analysis were predominantly developed and validated in Western, highly industrialized contexts. Accordingly, the process, trait, and environmental aspects we identified primarily reflect a WEIRD perspective on resilience. This is particularly true for the environmental category, in which the items focus on proximal social support (e.g. family and circle of friends) and individual resources in the immediate living environment, whereas macrostructural, cultural, or community‐related determinants (e.g. collective values, spirituality, and cultural narratives of coping) are only marginally represented or not represented at all. Although our framework is designed in principle to allow such culture‐ and context‐specific resources to be captured under the environmental or process dimension, the scales currently available make only limited use of this theoretical scope. Future work should therefore focus on developing and validating resilience measures that are anchored in different cultural and socioeconomic contexts and examine the extent to which the three‐level framework (trait, process, and environment) used here needs to be expanded to include explicit cultural and context‐related subdimensions.

Work should also continue to integrate resilience into the continuum of mental health and illness. In particular, models that follow a dimensional understanding of mental health and illness would enable this integration. The Hierarchical Taxonomy Of Psychopathology (HiTOP) model, for example, would be an interesting starting point in this regard. This could help to position resilience more precisely as an independent construct in the future or to use statistical modeling to contextualize it in relation to constructs such as vulnerability or personality dimensions.

### Conclusion

The aim of this study was to analyze how current psychometric instruments operationalize and measure psychological resilience and if the items of said instruments can be assigned to a resilience framework, derived from literature. Addressing this question is crucial as the concept of resilience is widely studied but often inconsistently defined, leading to potential confusion and dilution of the construct. The findings underscore the necessity of a clear and consistent approach to defining and measuring resilience to advance the field. The problem of different definitions and operationalizations of psychological resilience is also reflected within the psychometric instruments used to measure resilience.

The analysis of 83 resilience measures revealed a predominant focus on personality traits, with process‐oriented aspects and environmental factors receiving less attention. However, when adjusting for citation frequency, the emphasis shifted towards process‐oriented items, highlighting a discrepancy between the theoretical view of resilience as a dynamic process and the trait‐based focus of existing questionnaires. The categorization process was reliable, as indicated by a substantial inter‐rater agreement (κ = .68).

The findings suggest that the field of resilience research may benefit from greater clarity and specificity in its operationalization. To ensure that resilience remains a distinct and meaningful construct, future studies should prioritize transparent selection of scales and align these with clearly defined conceptual frameworks. This approach will not only improve the comparability of research findings but also safeguard the integrity of resilience as a construct, preventing its dilution and ensuring it retains its necessary discriminatory power.

## CONFLICT OF INTEREST STATEMENT

The authors declare that they have no competing interests. Open Access funding provided by Medizinische Universitat Graz.

## ETHICS STATEMENT

This study did not involve human participants, patient data, or any intervention. The analyses are based on published questionnaire items and anonymous expert ratings collected solely for methodological categorization (process/trait/environment and subdimensions). No personal, clinical, or otherwise identifiable information was obtained or processed. Under the applicable institutional and national guidelines for research ethics, projects that (a) use publicly available materials or syntactic reformulations thereof, (b) collect only non‐identifiable expert ratings about those materials, and (c) pose no foreseeable risk to individuals are exempt from ethics committee review. Accordingly, this work was not submitted to an ethics board. All expert raters were members of the research team or collaborators, informed about the study aims and procedures, and provided consent for their fully anonymized ratings to be analyzed and reported. No compensation, deception, or vulnerable populations were involved. The study complied with the principles of research integrity and data protection, and no individual rater can be identified.

## Supporting information


**Data S1.** Review articles on psychometric instruments used in resilience research

**Data S2.** Sensitivity analyses, including Rater 1

**Data S3.** Disagreed items – scale‐wise uncertainty, entropy, and confusion patterns

**Figure S1.** Framework for the rating process of stage I

**Figure S2.** Inter‐rater reliability and distribution of each category of the framework for each resilience questionnaire

**Figure S3.** Inter‐rater reliability and distribution of each category of the framework for each resilience questionnaire, with disagreement

**Table S1.** Resilience questionnaires and references

**Table S2.** Excluded resilience questionnaires, reference and reason for exclusion

**Table S3.** Percentage distribution of each category of the framework for each scale for raters 2–5 and items with reached agreed

## Data Availability

Further files, including the preregistration, the raw data, supplemental material, and the R syntax of the data analysis, can be accessed on the online repository of the OSF of this project (https://osf.io/6b7dg/).

## ENDNOTES

1 This is an easing factor for the raters because they always had to rate a block of items with the same instruction or the same clues and thus had to spend considerably less time reading additional information.

2 In this context, we use the absolute and not the relative citation frequency (citations divided by years since publication), as the aim is to compare the cumulative state of the literature.
