# Supplementary material for: Reverse‐engineering psychological resilience: A review and quantitative evaluation of psychometric instruments used in resilience research
Source: Appl Psychol Health Well Being. 2026 Jul 1;18(4):e70174. doi: 10.1111/aphw.70174 (PMC13321141; doi:10.1111/aphw.70174)
Supplement: Supplementary file 1 — Data S1. Review articles on psychometric instruments used in resilience research [file APHW-18-0-s007.docx]

1. **Supplement S2. Review articles on psychometric instruments used in resilience research**

Ahern, N. R., Kiehl, E. M., Lou Sole, M., & Byers, J. (2006). A review of instruments measuring resilience. *Issues in Comprehensive Pediatric Nursing*, *29*(2), 103–125. <https://doi.org/10.1080/01460860600677643>

Cosco, T. D., Kaushal, A., Richards, M., Kuh, D., & Stafford, M. (2016). Resilience measurement in later life: A systematic review and psychometric analysis. *Health and Quality of Life Outcomes*, *14*(1), 1-6. <https://doi.org/10.1186/s12955-016-0418-6>

Fisher, D. M., & Law, R. D. (2021). How to choose a measure of resilience: An organizing framework for resilience measurement. *Applied Psychology*, *70*(2), 643–673. <https://doi.org/10.1111/apps.12243>

Linden, B., Ecclestone, A., & Stuart, H. (2022). A scoping review and evaluation of instruments used to measure resilience among post-secondary students. *SSM - Population Health*, *19* (101227) 1-14. <https://doi.org/10.1016/j.ssmph.2022.101227>

Moore, T. M., White, L. K., Barzilay, R., Calkins, M. E., Jones, J. D., Young, J. F., Gur, R. C., & Gur, R. E. (2020). Development of a scale battery for rapid assessment of risk and resilience. *Psychiatry Research*, *288*(112996) 1-23. <https://doi.org/10.1016/j.psychres.2020.112996>

Palacio G, C., Krikorian, A., Gómez-Romero, M. J., & Limonero, J. T. (2020). Resilience in caregivers: A systematic review. *The American Journal of Hospice & Palliative Care*, *37*(8), 648–658. <https://doi.org/10.1177/1049909119893977>

Pangallo, A., Zibarras, L., Lewis, R., & Flaxman, P. (2015). Resilience through the lens of interactionism: A systematic review. *Psychological Assessment*, *27*(1), 1–20. <https://doi.org/10.1037/pas0000024>

Salisu, I., & Hashim, N. (2017). A critical review of scales used in resilience research. *IOSR Journal of Business and Management*. <https://doi.org/10.9790/487X-1904032333>

Satapathy, S., Dang, S., Sagar, R., & Dwivedi, S. N. (2022). Resilience in children and adolescents survived psychologically traumatic life events: A critical review of application of resilience assessment tools for clinical referral and intervention. *Trauma, Violence, & Abuse*, *23*(1), 1–13. <https://doi.org/10.1177/1524838020939126>

Seiler, A., & Jenewein, J. (2019). Resilience in cancer patients. *Frontiers in Psychiatry*, *10*(208) 1-35. <https://doi.org/10.3389/fpsyt.2019.00208>

Seko, Y., De-Lawrence, L., Nalder, E., & King, G. (2020). Assessing resiliency in pediatric rehabilitation: A critical review of assessment tools and applications. *Child: Care, Health and Development*, *46*, 249–267. <https://doi.org/10.1111/cch.12743>

Smith-Osborne, A., & Whitehill Bolton, K. (2013). Assessing resilience: A review of measures across the life course. *Journal of Evidence-Based Social Work*, *10*(2), 111–126. <https://doi.org/10.1080/15433714.2011.597305>

Vannest, K. J., Ura, S. K., Lavadia, C., & Zolkoski, S. (2021). Self-report measures of resilience in children and youth. *Contemporary School Psychology*, *25*(4), 406–415. <https://doi.org/10.1007/s40688-019-00252-1>

Wadi, M., Nordin, N. I., Roslan, N. S., Tan, C., & Yusoff, M. (2020). Reframing resilience concept: Insights from a meta-synthesis of 21 resilience scales. *Education in Medicine Journal, 12*(2), 3-22. <https://doi.org/10.21315/eimj2020.12.2.2>

Windle, G., Bennett, K. M., & Noyes, J. (2011). A methodological review of resilience measurement scales. *Health and Quality of Life Outcomes*, *9*(8), 1-18. <https://doi.org/10.1186/1477-7525-9-8>

Zhou, J., He, B., He, Y., Huang, W., Zhu, H., Zhang, M., & Wang, Y. (2020). Measurement properties of family resilience assessment questionnaires: A systematic review. *Family Practice*, *37*(5), 581–591. <https://doi.org/10.1093/fampra/cmaa027>

Zolkoski, S. M., & Bullock, L. M. (2012). Resilience in children and youth: A review. *Children and Youth Services Review*, *34*(12), 2295–2303. <https://doi.org/10.1016/j.childyouth.2012.08.009>
