# Supplementary material for: Reverse‐engineering psychological resilience: A review and quantitative evaluation of psychometric instruments used in resilience research
Source: Appl Psychol Health Well Being. 2026 Jul 1;18(4):e70174. doi: 10.1111/aphw.70174 (PMC13321141; doi:10.1111/aphw.70174)
Supplement: Supplementary file 2 — Data S2. Sensitivity analyses, including Rater 1 [file APHW-18-0-s008.docx]

# Supplement S3. Sensitivity analyses, including Rater 1

## Objective and approach

To test robustness, we repeated the categorization of items once with the original four-rater panel including rater 1 (R1–R4; majority decision ≥ 3/4) and compared the results with the final panel without rater 1 (R2–R5; majority decision ≥ 3/4). We analyzed (i) the unweighted and citation-weighted distributions of the three primary categories process, trait, and environment, and (ii) the corresponding distributions at the subdimension level (resilience, vulnerability, posttraumatic growth, resistance; the Big Five factors; social support and other non-social factors). For both panel variants, mean values, citation-weighted mean values (weighting: citation frequency of the scale), and the weighting-related difference ($\Delta$= weighted - unweighted) were calculated. Additionally, we examined rankings (e.g., citation-weighted proportions) and their changes.

## Results

Primary categories (process/trait/environment). The distributions were highly similar between R1–R4 and R2–R5. The citation-weighted proportions differed only slightly (all deviations within a small range, with a maximum absolute deviation of approximately 3.78%), with no shifts in the overall patterns. The ranking of the three top-level categories remained stable (average rank change ≈ 0.33; max. rank change = 1).

At the subdimension level, differences between panels were minimal, with an average |$\Delta$| of citation-weighted shares around 0.81% and a maximum of 4.35%. Some subdimensions experienced slight ranking changes (with an average rank change of 0.27), but these did not alter the overall dominance pattern.

The sensitivity analysis shows that the inclusion or exclusion of rater 1 does not substantially change either the central distributions at the P/T/E level or the subdimensional patterns while simultaneously improving homogeneity (interrater agreement) from a kappa coefficient of $\kappa$ = .61 to $\kappa$ = .68. All primary statements remain unchanged.

Table S3.1. Sensitivity analysis of the main levels (Process/Trait/Environment) between panels R1–R4 and R2–R5.

| Level | 1 | 2 | 3 | 4 | 5 | 6 | 7 | 8 | 9 | 10 | 11 | 12 |
| --- | --- | --- | --- | --- | --- | --- | --- | --- | --- | --- | --- | --- |
| Process | 31.79 | 30.14 | -1.65 | 60.17 | 56.39 | -3.78 | 28.38 | 26.25 | -2.13 | 1 | 1 | 0 |
| Trait | 51.95 | 51.62 | -0.33 | 34.59 | 37.85 | 3.26 | -17.36 | -13.77 | 3.59 | 3 | 2 | -1 |
| Environment | 16.26 | 18.24 | 1.98 | 5.24 | 5.76 | 0.52 | -11.02 | -12.49 | -1.47 | 8 | 8 | 0 |

*Note*. Mean rater 2-5 (1), mean rater 1-4 (2), difference mean (3), weighted mean rater 2-5 (4), weighted mean rater 1-4 (5), difference weighted mean (6), delta weight rater 2-5 (7), delta weight rater 1-4 (8), difference delta weight (9), rank rater 2-5 (10), rank rater 1-4 (11), rank change (12).

Table S3.2. Sensitivity analysis of all dimensions between panels R1–R4 and R2–R5.

| Dimension | 1 | 2 | 3 | 4 | 5 | 6 | 7 | 8 | 9 | 10 | 11 | 12 |
| --- | --- | --- | --- | --- | --- | --- | --- | --- | --- | --- | --- | --- |
| Resilience | 23.06 | 22.36 | -0.7 | 36.03 | 36.41 | 0.38 | 12.98 | 14.05 | 1.07 | 2 | 3 | 1 |
| Vulnerability | 0.04 | 0.04 | 0 | 0.02 | 0.02 | 0 | -0.02 | -0.02 | 0 | 14 | 14 | 0 |
| Posttraumatic Growth | 7.43 | 6.42 | -1.01 | 23.13 | 18.78 | -4.35 | 15.7 | 12.36 | -3.34 | 4 | 4 | 0 |
| Resistance | 1.26 | 1.32 | 0.06 | 0.99 | 1.18 | 0.19 | -0.28 | -0.14 | 0.14 | 12 | 12 | 0 |
| Openness | 10.78 | 10.69 | -0.09 | 10.38 | 10.94 | 0.56 | -0.4 | 0.26 | 0.66 | 5 | 5 | 0 |
| Conscientiousness | 14.86 | 15.35 | 0.49 | 9.21 | 10.76 | 1.55 | -5.65 | -4.59 | 1.06 | 6 | 6 | 0 |
| Extraversion | 9.36 | 8.98 | -0.38 | 6.13 | 6 | -0.13 | -3.23 | -2.98 | 0.25 | 7 | 7 | 0 |
| Agreeableness | 9.45 | 9.05 | -0.4 | 4.69 | 5.4 | 0.71 | -4.75 | -3.65 | 1.1 | 10 | 9 | -1 |
| Neuroticism | 7.51 | 7.55 | 0.04 | 4.19 | 4.75 | 0.56 | -3.33 | -2.8 | 0.53 | 11 | 11 | 0 |
| Social Factors - Social Support | 14.22 | 15.67 | 1.45 | 4.89 | 5.37 | 0.48 | -9.33 | -10.3 | -0.97 | 9 | 10 | 1 |
| Other non-social Factors | 2.04 | 2.57 | 0.53 | 0.35 | 0.38 | 0.03 | -1.69 | -2.18 | -0.49 | 13 | 13 | 0 |

*Note*. Mean rater 2-5 (1), mean rater 1-4 (2), difference mean (3), weighted mean rater 2-5 (4), weighted mean rater 1-4 (5), difference weighted mean (6), delta weight rater 2-5 (7), delta weight rater 1-4 (8), difference delta weight (9), rank rater 2-5 (10), rank rater 1-4 (11), rank change (12).
