# Supplementary material for: Reverse‐engineering psychological resilience: A review and quantitative evaluation of psychometric instruments used in resilience research
Source: Appl Psychol Health Well Being. 2026 Jul 1;18(4):e70174. doi: 10.1111/aphw.70174 (PMC13321141; doi:10.1111/aphw.70174)
Supplement: Supplementary file 3 — Data S3. Disagreed items – scale‐wise uncertainty, entropy, and confusion patterns [file APHW-18-0-s003.docx]

# Supplement S4. Disagreed items – scale-wise uncertainty, entropy, and confusion patterns

## Objective and approach

This supplement documents a systematic additional analysis of items without majority consensus (panels R2–R5). It reports (a) disagreement rates per scale with robust 95% Wilson confidence intervals, (b) the Shannon entropy of the subdimension judgments as a measure of conceptual heterogeneity (all items vs. only disagreed items), and (c) pairwise confusion matrices at the level of the three main categories (Process/Trait/Environment) and the subdimensions.

First, we calculated two measures of uncertainty (panels R2–R5) for each scale:

- Disagreement rate: proportion of items without majority consensus (≥3/4 raters). We also report 95% Wilson confidence intervals for these proportions (robust for small n).
- Shannon entropy of the subdimension ratings per item as a measure of conceptual heterogeneity (higher = wider distribution, less focused assessments) We provide the mean entropy across all items and across disagreed items only for each scale.

Furthermore, we generated a confusion matrix for all rated items. For each item, all unordered rater pairs were formed from the 4-person panel (with 4 raters, there are 6 pairs per item). Each pair contributes exactly one observation to the confusion matrix:

- Agreement: Both ratings in the pair have the same category (e.g., Process vs. Process or Resilience vs. Resilience). These cases lie on the main diagonal of the matrix.
- Disagreement: The pair shows two different categories (e.g., Process vs. Trait or Resilience vs. Neuroticism). These cases lie outside the diagonal.

We count unordered pairs. A pair {A, B} is the same as {B, A}. Therefore, the matrices are symmetric about the diagonal, and the entries for A–B and B–A are identical. For this reason, we report only one half of the matrix (upper triangle) in the figures to avoid redundancy. The percentages indicate the proportion of a cell relative to the total number of all rated pairs across all items.

## Results

The median disagreement rate across all scales (N = 83) is 26.09% (IQR: 14.67%–36.18%; range: 0%–73.33%). The mean entropy is 0.38 (all items) and 1.02 (disagreed items only), i.e., as expected, ambiguities are associated with greater heterogeneity in ratings.

Examples of the highest disagreement rates are:

- ARS-30: 73.33% disagreed; n = 30; entropy: 0.86 (all items), 1.17 (disagreed only).
- MTRR-99: 59.60% disagreed; n = 99; entropy: 0.79 (all items), 1.11 (disagreed only).
- RAU: 57.89% disagreed; n = 19; entropy: 0.72 (all items), 1.04 (disagreed only).

8 scales are at ≥ 50% disagreement (higher conceptual ambiguity). 21 scales are at ≤ 15% disagreement (more robust classification), of which 18 scales are at ≤ 10%; 13 scales have 0% disagreement. An overview of all values per scale can be found in Supplemental Table S4.1.

The pairwise confusion analysis shows that, across the three main rating levels, the most common mismatch is Process ↔ Trait. Specifically:

- 10.67% of all pair comparisons are Process vs. Trait (and mirrored),
- 6.88% on trait vs. environment,
- 1.38% on process vs. environment

This means that the greatest confusion is located at the process/trait interface, while environment is less frequently confused with the other levels (for further information, see Supplemental Table S4.2).

A similar picture emerges for the subdimensions: more frequent confusions involve pairs that are conceptually close to process or trait aspects. The highest off-diagonal pairs (excerpt) are:

- Resilience ↔ Neuroticism: 2.36%,
- Resilience ↔ Resistance: 2.29%,
- Agreeableness ↔ Social Support: 2.15%,
- Conscientiousness ↔ Neuroticism: 1.93%,
- Extraversion ↔ Agreeableness: 1.69%

These patterns demonstrate systematic borderline situations (e.g., between resilient process characteristics and trait-related negative affect dimensions) as well as socially embedded confusions (Agreeableness ↔ Social Support). Please refer to Supplemental Table S4.3 for further information.

Confusion matrices (Supplemental Figure S4.1 [Level] and Supplemental Figure S4.2 [Subdimensions]) visualize the results above. The main diagonal aggregates all pairwise matches (two raters of the same item chose the same category). In the percentage representation, the diagonal serves as a reference for how often rater pairs categorize items the same way – the off-diagonal proportions, on the other hand, quantify ambiguity.

## Discussion

The scale-by-scale uncertainty analysis directly addresses the question of whether excluding disagreed items could distort the findings. The following points are crucial: Some scales (e.g., ARS-30, MTRR-99, RAU) show higher disagreement rates and entropies as expected; this is plausible because their items more frequently contain wordings that operate at process/trait boundaries. Other scales (e.g., STARS, BRCS, BRS) are more conceptually focused; here, ratings are more consistent. These differences highlight scale-specific details but do not alter the overall P/T/E pattern in the primary analyses. The higher entropy of the disagreed items indicates conceptual ambiguity; these items were consistently excluded by the main approach (majority criterion). S4.1 at the scale level provides clear reference values (proportions and entropies) for readers who require detailed information. Higher disagreement rates should be reflected in relation to the scale without relativizing the overall interpretation.

The pairwise confusion analyses show where and to what extent ambiguities occur, without assuming a “gold standard” category. The off-diagonal percentages are concentrated in a few theoretically plausible border areas – primarily Process ↔ Trait (10.67%) and, in the subdimension area, at interfaces between process-related resilience facets and trait-related negative affect or socially influenced dimensions. In contrast, environment confusions are considerably lower (e.g., Process ↔ Environment 1.38%). This does not indicate lack of clarity in the framework overall, but rather specific transition zones that are conceptually expected.

Figure S4.1. Confusion Matrix – Main Levels (P/T/E) 3×3

*Note*. The figure shows the percentages of all unordered pairwise rater comparisons (R2–R5) at the process/trait/environment level; only the upper half of the triangle (including the diagonal) is shown, as A–B and B–A are combined. The diagonal indicates agreements between rater pairs, while off-diagonals mark systematic confusions between two levels. The values are scaled to 100% across all cells in the upper half of the triangle (including the diagonal).

Figure S4.2. Confusion Matrix – Subdimensions 12×12

*Note*. The percentages of all unsorted pairwise rater comparisons (R2–R5) across 12 subdimensions are shown; only the upper half of the triangle (including the diagonal) is displayed, as A–B and B–A are combined. The diagonal represents agreement within a subdimension, while off-diagonals indicate frequent borderline cases/confusions between two subdimensions. Percentages add up to 100% across the upper half of the triangle (including the diagonal).

Table S4.1. Scale-wise ambiguity indices (disagreement rate with Wilson CI and entropy)

| Scale | Items (N) | Disagreed (%) | 95% CI  (Wilson) | Entropy (all) | Entropy  (disagreed) |
| --- | --- | --- | --- | --- | --- |
| ARS-30 | 30 | 73.3 | [55.6; 85.8] | 0.86 | 1.17 |
| MTRR-99 | 99 | 59.6 | [49.7; 68.7] | 0.79 | 1.11 |
| RAU | 19 | 57.9 | [36.3; 76.9] | 0.72 | 1.04 |
| PRS | 15 | 53.3 | [30.1; 75.2] | 0.80 | 1.08 |
| RSCA | 64 | 53.1 | [41.1; 64.8] | 0.69 | 1.07 |
| RASS | 19 | 52.6 | [31.7; 72.7] | 0.61 | 1.11 |
| PCQ-12 | 12 | 50 | [25.4; 74.6] | 0.66 | 1.04 |
| PR6-16 | 16 | 50 | [28.0; 72.0] | 0.57 | 1.00 |
| MeRS | 37 | 48.6 | [33.4; 64.1] | 0.58 | 1.04 |
| FRA | 29 | 48.3 | [31.4; 65.6] | 0.67 | 1.06 |
| CYRM-R (Youth) | 17 | 47.1 | [26.2; 69.0] | 0.54 | 1.08 |
| PCQ | 24 | 45.8 | [27.9; 64.9] | 0.58 | 1.10 |
| ER | 29 | 44.8 | [28.4; 62.5] | 0.51 | 0.88 |
| RSYA | 50 | 44 | [31.2; 57.7] | 0.55 | 0.96 |
| ARM-R | 17 | 41.2 | [21.6; 64.0] | 0.44 | 0.99 |
| BURS | 30 | 40 | [24.6; 57.7] | 0.56 | 0.92 |
| RASP | 34 | 38.2 | [23.9; 55.0] | 0.48 | 1.12 |
| PR6-50 | 50 | 38 | [25.9; 51.8] | 0.48 | 1.00 |
| ERESMA | 45 | 37.8 | [25.1; 52.4] | 0.52 | 1.12 |
| RSAS | 45 | 37.8 | [25.1; 52.4] | 0.52 | 0.98 |
| RSES | 22 | 36.4 | [19.7; 57.0] | 0.52 | 1.00 |
| CD-RISC-25 | 25 | 36 | [20.2; 55.5] | 0.39 | 1.08 |
| RRC-ARM | 28 | 35.7 | [20.7; 54.2] | 0.59 | 1.14 |
| FRI | 20 | 35 | [18.1; 56.7] | 0.53 | 1.19 |
| PFRS | 20 | 35 | [18.1; 56.7] | 0.40 | 0.99 |
| IFCR | 75 | 34.7 | [24.9; 45.9] | 0.59 | 1.11 |
| DRS-15 | 15 | 33.3 | [15.2; 58.3] | 0.54 | 1.18 |
| DRS-45 | 45 | 33.3 | [21.4; 47.9] | 0.51 | 1.16 |
| RS | 25 | 32 | [17.2; 51.6] | 0.41 | 1.08 |
| FRAS | 67 | 31.3 | [21.5; 43.2] | 0.43 | 1.19 |
| FRS16 | 16 | 31.2 | [14.2; 55.6] | 0.42 | 1.11 |
| CD-RISC-10 | 10 | 30 | [10.8; 60.3] | 0.31 | 1.04 |
| DRS-30 | 30 | 30 | [16.7; 47.9] | 0.48 | 1.16 |
| CYRM-R (Child) | 17 | 29.4 | [13.3; 53.1] | 0.39 | 1.11 |
| RAS | 24 | 29.2 | [14.9; 49.2] | 0.41 | 1.09 |
| 7C | 7 | 28.6 | [8.2; 64.1] | 0.46 | 1.04 |
| RS-14 | 14 | 28.6 | [11.7; 54.6] | 0.34 | 1.04 |
| WRI | 60 | 28.3 | [18.5; 40.8] | 0.38 | 1.06 |
| WFRQ | 32 | 28.1 | [15.6; 45.4] | 0.44 | 1.08 |
| RPFC | 25 | 28 | [14.3; 47.6] | 0.35 | 1.09 |
| ER-11 | 11 | 27.3 | [9.7; 56.6] | 0.30 | 0.92 |
| DARS | 23 | 26.1 | [12.5; 46.5] | 0.34 | 0.92 |
| CHKS | 47 | 25.5 | [15.3; 39.5] | 0.44 | 1.07 |
| MMPR | 40 | 25 | [14.2; 40.2] | 0.30 | 0.90 |
| R-MATS | 24 | 25 | [12.0; 44.9] | 0.35 | 1.10 |
| RSA (2005) | 33 | 24.2 | [12.8; 41.0] | 0.34 | 1.04 |
| ARQ | 88 | 23.9 | [16.2; 33.7] | 0.32 | 1.17 |
| ER89 | 14 | 21.4 | [7.6; 47.6] | 0.32 | 0.92 |
| RESI-M | 43 | 20.9 | [11.4; 35.2] | 0.26 | 0.89 |
| TRS-C | 64 | 20.3 | [12.3; 31.7] | 0.33 | 0.96 |
| ERS-15 | 15 | 20 | [7.0; 45.2] | 0.30 | 0.92 |
| RAQ-40 | 40 | 20 | [10.5; 34.8] | 0.23 | 0.95 |
| RS-10 | 10 | 20 | [5.7; 51.0] | 0.29 | 0.87 |
| RS-5 | 5 | 20 | [3.6; 62.4] | 0.32 | 1.04 |
| RSA (2003) | 36 | 19.4 | [9.8; 35.0] | 0.26 | 0.94 |
| ARS | 21 | 19 | [7.7; 40.0] | 0.22 | 0.87 |
| HCRS | 16 | 18.8 | [6.6; 43.0] | 0.37 | 1.04 |
| MIIRM | 22 | 18.2 | [7.3; 38.5] | 0.42 | 1.04 |
| FRS-V | 6 | 16.7 | [3.0; 56.4] | 0.17 | 1.04 |
| RSS | 12 | 16.7 | [4.7; 44.8] | 0.17 | 1.04 |
| TRAS | 12 | 16.7 | [4.7; 44.8] | 0.24 | 0.87 |
| 5x5RS | 25 | 16 | [6.4; 34.7] | 0.26 | 1.04 |
| BPFI | 15 | 13.3 | [3.7; 37.9] | 0.15 | 0.87 |
| RAQ-8 | 8 | 12.5 | [2.2; 47.1] | 0.20 | 1.04 |
| SPF | 24 | 12.5 | [4.3; 31.0] | 0.16 | 0.92 |
| ER89-R | 10 | 10 | [1.8; 40.4] | 0.27 | 1.04 |
| RS-11 | 11 | 9.1 | [1.6; 37.7] | 0.20 | 1.04 |
| SEARS-C | 35 | 5.7 | [1.6; 18.6] | 0.24 | 0.87 |
| SEARS-T | 41 | 4.9 | [1.3; 16.1] | 0.14 | 0.87 |
| SEARS-A | 35 | 2.9 | [0.5; 14.5] | 0.26 | 0.69 |
| BRCS | 4 | 0 | [0.0; 49.0] | 0.141 | NA |
| BRS | 6 | 0 | [-0.0; 39.0] | 0 | NA |
| CD-RISC-2 | 2 | 0 | [0.0; 65.8] | 0 | NA |
| HGRS | 9 | 0 | [0.0; 29.9] | 0.062 | NA |
| PTGI | 21 | 0 | [0.0; 15.5] | 0.161 | NA |
| PTGI-SF | 10 | 0 | [-0.0; 27.8] | 0.169 | NA |
| PTGI-X | 25 | 0 | [0.0; 13.3] | 0.157 | NA |
| SEARS-A-S | 12 | 0 | [-0.0; 24.2] | 0.141 | NA |
| SEARS-C-S | 12 | 0 | [-0.0; 24.2] | 0.187 | NA |
| SEARS-P | 39 | 0 | [0.0; 9.0] | 0.288 | NA |
| SEARS-P-S | 12 | 0 | [-0.0; 24.2] | 0.187 | NA |
| SEARS-T-S | 12 | 0 | [-0.0; 24.2] | 0.094 | NA |
| STARS | 13 | 0 | [0.0; 22.8] | 0.476 | NA |

*Note*. Each line corresponds to a scale. Items (N) = number of items rated; Disagreed (N) = number of items without a 3/4 majority; Disagreed (%) = proportion of ambiguous items; 95% CI (Wilson) = upper / lower CI: 95% Wilson confidence interval of disagreement rate; Entropy (all) = mean Shannon entropy across all items; Entropy (disagreed) = mean entropy only across disagreed items. Higher entropy indicates broader distributions of judgments (greater ambiguity). Percentages rounded to two decimal places where applicable.

Table S4.2. Rating Confusions – Main Levels (P/T/E)

| Level A | Level B | Percent |
| --- | --- | --- |
| Process | Trait | 10.67 |
| Trait | Environment | 6.88 |
| Process | Environment | 1.38 |

Table S4.3. Rating Confusions – Subdimensions

| Subdimension A | Subdimension B | Percent |
| --- | --- | --- |
| Resilience | Neuroticism | 2.36 |
| Resilience | Resistance | 2.29 |
| Agreeableness | Social Factors - Social Support | 2.15 |
| Conscientiousness | Neuroticism | 1.93 |
| Extraversion | Agreeableness | 1.69 |
| Resilience | Posttraumatic Growth | 1.52 |
| Resilience | Conscientiousness | 1.49 |
| Resistance | Neuroticism | 1.37 |
| Extraversion | Neuroticism | 1.27 |
| Extraversion | Social Factors - Social Support | 1.24 |
| Openness | Conscientiousness | 1.23 |
| Resilience | Openness | 1.20 |
| Social Factors - Social Support | Other non-social Factors | 0.96 |
| Resistance | Conscientiousness | 0.82 |
| Resilience | Social Factors - Social Support | 0.81 |
| Conscientiousness | Other non-social Factors | 0.74 |
| Openness | Extraversion | 0.73 |
| Conscientiousness | Extraversion | 0.72 |
| Resilience | Extraversion | 0.66 |
| Openness | Neuroticism | 0.63 |
| Openness | Agreeableness | 0.62 |
| Resilience | Agreeableness | 0.59 |
| Neuroticism | Other non-social Factors | 0.59 |
| Agreeableness | Neuroticism | 0.56 |
| Agreeableness | Other non-social Factors | 0.55 |
| Conscientiousness | Agreeableness | 0.49 |
| Openness | Other non-social Factors | 0.43 |
| Openness | Social Factors - Social Support | 0.39 |
| Neuroticism | Social Factors - Social Support | 0.30 |
| Posttraumatic Growth | Openness | 0.29 |
| Extraversion | Other non-social Factors | 0.27 |
| Posttraumatic Growth | Conscientiousness | 0.26 |
| Resilience | Other non-social Factors | 0.25 |
| Posttraumatic Growth | Neuroticism | 0.24 |
| Resistance | Openness | 0.22 |
| Conscientiousness | Social Factors - Social Support | 0.22 |
| Resilience | Vulnerability | 0.21 |
| Vulnerability | Neuroticism | 0.21 |
| Resistance | Extraversion | 0.18 |
| Posttraumatic Growth | Social Factors - Social Support | 0.16 |
| Resistance | Agreeableness | 0.13 |
| Posttraumatic Growth | Resistance | 0.12 |
| Posttraumatic Growth | Extraversion | 0.11 |
| Vulnerability | Agreeableness | 0.11 |
| Vulnerability | Conscientiousness | 0.11 |
| Vulnerability | Openness | 0.07 |
| Vulnerability | Extraversion | 0.07 |
| Posttraumatic Growth | Agreeableness | 0.05 |
| Resistance | Social Factors - Social Support | 0.05 |
| Vulnerability | Resistance | 0.05 |
| Posttraumatic Growth | Other non-social Factors | 0.05 |
| Vulnerability | Social Factors - Social Support | 0.04 |
| Vulnerability | Other non-social Factors | 0.04 |
| Vulnerability | Posttraumatic Growth | 0.02 |
| Resistance | Other non-social Factors | 0.01 |
