# Supplementary material for: Reverse‐engineering psychological resilience: A review and quantitative evaluation of psychometric instruments used in resilience research
Source: Appl Psychol Health Well Being. 2026 Jul 1;18(4):e70174. doi: 10.1111/aphw.70174 (PMC13321141; doi:10.1111/aphw.70174)
Supplement: Supplementary file 5 — Figure S2. Inter‐rater reliability and distribution of each category of the framework for each resilience questionnaire [file APHW-18-0-s006.docx]

Figure S2. Inter-rater reliability and distribution of each category of the framework for each resilience questionnaire
