# Supplementary material for: Reverse‐engineering psychological resilience: A review and quantitative evaluation of psychometric instruments used in resilience research
Source: Appl Psychol Health Well Being. 2026 Jul 1;18(4):e70174. doi: 10.1111/aphw.70174 (PMC13321141; doi:10.1111/aphw.70174)
Supplement: Supplementary file 7 — Table S1. Resilience questionnaires and references [file APHW-18-0-s004.docx]

Table S1. Resilience questionnaires and references

| Abbreviation | Reference |
| --- | --- |
| 5×5RS | DeSimone, J. A., Harms, P. D., Vanhove, A. J., & Herian, M. N. (2017). Development and validation of the Five-by-Five Resilience Scale. *Assessment*, *24*(6), 778–797. <https://doi.org/10.1177/1073191115625803> |
| 7C | Barger, J., Vitale, P., Gaughan, J. P., & Feldman-Winter, L. (2017). Measuring resilience in the adolescent population: A succinct tool for outpatient adolescent health. *The Journal of Pediatrics*, *189*, 201-206. <https://doi.org/10.1016/j.jpeds.2017.06.030> |
| ARM-R | http://www.resilienceresearch.org/ |
| ARQ | Gartland, D., Bond, L., Olsson, C. A., Buzwell, S., & Sawyer, S. M. (2011). Development of a multi-dimensional measure of resilience in adolescents: The Adolescent Resilience Questionnaire. *BMC Medical Research Methodology*, *11*(1), 134-144. <https://doi.org/10.1186/1471-2288-11-134> |
| ARS | Oshio, A., Kaneko, H., Nagamine, S., & Nakaya, M. (2003). Construct validity of the Adolescent Resilience Scale. *Psychological Reports*, *93*, 1217–1222. <https://doi.org/10.2466/pr0.2003.93.3f.1217> |
| ARS-30 | Cassidy, S. (2016). The Academic Resilience Scale (ARS-30): A new multidimensional construct measure. *Frontiers in Psychology*, *7, 1-11*. <https://doi.org/10.3389/fpsyg.2016.01787> |
| BPFI | Baruth, K. E., & Caroll, J. J. (2002). A formal assessment of resilience: The Baruth Protective Factors Inventory. *The Journal of Individual Psychology, 58*(3), 235–244. |
| BRCS | Sinclair, V. G., & Wallston, K. A. (2004). The development and psychometric evaluation of the Brief Resilient Coping Scale. *Assessment*, *11*(1), 94–101. <https://doi.org/10.1177/1073191103258144> |
| BRS | Smith, B. W., Dalen, J., Wiggins, K., Tooley, E., Christopher, P., & Bernard, J. (2008). The brief resilience scale: Assessing the ability to bounce back. *International Journal of Behavioral Medicine*, *15*(3), 194–200. <https://doi.org/10.1080/10705500802222972> |
| BURS | Annalakshmi N. (2009) Bharathiar University resilience scale. In H. Purohit & A. Wagh (Eds.), *Research Methodology Tools and Techniques.* Sri Publishers, 105–121. |
| CD-RISC-10 | Campbell‐Sills, L., & Stein, M. B. (2007). Psychometric analysis and refinement of the connor–davidson resilience scale (CD‐RISC): Validation of a 10‐item measure of resilience. *Journal of Traumatic Stress*, *20*(6), 1019–1028. <https://doi.org/10.1002/jts.20271> |
| CD-RISC-2 | Vaishnavi, S., Connor, K., & Davidson, J. R. T. (2007). An abbreviated version of the Connor-Davidson Resilience Scale (CD-RISC), the CD-RISC2: Psychometric properties and applications in psychopharmacological trials. *Psychiatry Research*, *152*(2–3), 293–297. <https://doi.org/10.1016/j.psychres.2007.01.006> |
| CD-RISC-25 | Connor, K. M., & Davidson, J. R. T. (2003). Development of a new resilience scale: The Connor-Davidson Resilience Scale (CD-RISC). *Depression and Anxiety*, *18*(2), 76–82. <https://doi.org/10.1002/da.10113> |
| CHKS | Hanson, T. L., & Kim, J. O. (2007). Measuring resilience and youth development: the psychometric properties of the Healthy Kids Survey. |
| CYRM-R (Child) | Jefferies, P., McGarrigle, L., & Ungar, M. (2019). The CYRM-R: A Rasch-Validated Revision of the Child and Youth Resilience Measure. *Journal of Evidence-Based Social Work*, *16*(1), 70–92. <https://doi.org/10.1080/23761407.2018.1548403> |
| CYRM-R (Youth) | Jefferies, P., McGarrigle, L., & Ungar, M. (2019). The CYRM-R: A Rasch-Validated Revision of the Child and Youth Resilience Measure. *Journal of Evidence-Based Social Work*, *16*(1), 70–92. <https://doi.org/10.1080/23761407.2018.1548403> |
| DARS | Mackrain, M. (2007). *Devereux Adult Resilience Survey*. The Devereux Foundation. |
| DRS-15 | Bartone, P. T., Ursano, R. J., Wright, K. M., & Ingraham, L. H. (1989). The Impact of a Military Air Disaster on The Health of Assistance Workers: A Prospective Study. *The Journal of Nervous and Mental Disease*, *177*(6), 317–328. <https://doi.org/10.1097/00005053-198906000-00001> |
| DRS-30 | Bartone, P. T. (1991). Development and validation of a short hardiness measure. In *annual convention of the American Psychological Society. Washington DC*. |
| DRS-45 | Bartone, P. T., Ursano, R. J., Wright, K. M., & Ingraham, L. H. (1989). The impact of a military air disaster on the health of assistance workers: A prospective study. *The Journal of Nervous and Mental Disease*, *177*(6), 317–328. <https://doi.org/10.1097/00005053-198906000-00001> |
| ER | Klohnen, E. C. (1996). Conceptual analysis and measurement of the construct of ego-resiliency. *Journal of Personality and Social Psychology*, *70*(5), 1067–1079. <https://doi.org/10.1037/0022-3514.70.5.1067> |
| ER-11 | Farkas, D., & Orosz, G. (2015). Ego-resiliency reloaded: A three-component model of general resiliency. *PLOS ONE*, *10*(3), 1-26. <https://doi.org/10.1371/journal.pone.0120883> |
| ER89 | Block, J., & Kremen, A. M. (1996). IQ and ego-resiliency: Conceptual and empirical connections and separateness. *Journal of Personality and Social Psychology*, *70*(2), 349–361. <https://doi.org/10.1037/0022-3514.70.2.349> |
| ER89-R | Alessandri, G., Vecchione, M., Caprara, G., & Letzring, T. D. (2012). The Ego Resiliency Scale revised: A crosscultural study in Italy, Spain, and the United States. *European Journal of Psychological Assessment*, *28*(2), 139–146. <https://doi.org/10.1027/1015-5759/a000102> |
| ERESMA | Roque‚ M. P.‚ Acle‚ G., & García‚ M. (2009). Escala de resilienciamaterna: Un estudio de validación en una muestra de madres con niños especiales [Maternal resilience scale: A validation study in a sample of mothers with exceptional children]. *Revista Iberoamericana de Diagnóstico y Evaluación Psicológica‚ 1*(27)‚ 107–132. |
| ERS-15 | Chen, X., Wang, Y., & Yan, Y. (2016). The Essential Resilience Scale: Instrument development and prediction of perceived health and behaviour. *Stress and Health*, *32*(5), 533–542. <https://doi.org/10.1002/smi.2659> |
| FRA | Duncan Lane, C., Meszaros, P. S., & Savla, J. (2017). Measuring Walsh’s family resilience framework: Reliability and validity of the family resilience assessment among women with a history of breast cancer. *Marriage & Family Review*, *53*(7), 667–682. <https://doi.org/10.1080/01494929.2016.1263588> |
| FRAS | Sixbey‚ M.‚ T. (2005). *Development of the family resilience assessment scale to identify family resilience constructs.*[Doctoral dissertation, University of Florida]. [https://ufdc.ufl.edu/ufe0012882/00001](https://ufdc.ufl.edu/UFE0012882/00001/images) |
| FRI | Burnette, C. E., Boel‐Studt, S., Renner, L. M., Figley, C. R., Theall, K. P., Miller Scarnato, J., & Billiot, S. (2020). The Family Resilience Inventory: A culturally grounded measure of current and family‐of‐origin protective processes in native American families. *Family Process*, *59*(2), 695–708. <https://doi.org/10.1111/famp.12423> |
| FRS-16 | Chow, T. S., Tang, C. S. K., Siu, T. S. U., & Kwok, H. S. H. (2022). Family Resilience Scale Short Form (FRS16): Validation in the US and Chinese Samples. *Frontiers in Psychiatry*, *13*(845803) 1-12. <https://doi.org/10.3389/fpsyt.2022.845803> |
| FRS-V | Finley, E. P., Pugh, M. J., & Palmer, R. F. (2016). Validation of a measure of family resilience among Iraq and Afghanistan veterans. *Military Behavioral Health*, *4*(3), 205–219. <https://doi.org/10.1080/21635781.2016.1153530> |
| HCRS | Joinson, A. N., Dixon, M., Coventry, L., & Briggs, P. (2023). Development of a new ‘human cyber-resilience scale.’ *Journal of Cybersecurity*, *9*(1), 1-10. <https://doi.org/10.1093/cybsec/tyad007> |
| HGRS | Hardy, S. E., Concato, J., & Gill, T. M. (2004). Resilience of community‐dwelling older persons. *Journal of the American Geriatrics Society*, *52*(2), 257–262. <https://doi.org/10.1111/j.1532-5415.2004.52065.x> |
| IFCR | Distelberg, B. J., Martin, A. S., Borieux, M., & Oloo, W. A. (2015). Multidimensional family resilience assessment: The Individual, Family, and Community Resilience (IFCR) Profile. *Journal of Human Behavior in the Social Environment*, *25*(6), 552–570. <https://doi.org/10.1080/10911359.2014.988320> |
| MeRS | Rahman, M. A., Yusoff, M. S. B., Roslan, N. S., Mohammad, J. A.-M., & Ahmad, A. (2021). Development and validation of the medical professionals resilience scale. *BMC Health Services Research*, *21*(482), 1-9. <https://doi.org/10.1186/s12913-021-06542-w> |
| MIIRM | Martin, A. S., Distelberg, B., Palmer, B. W., & Jeste, D. V. (2015). Development of a new multidimensional individual and interpersonal resilience measure for older adults. *Aging & Mental Health*, *19*(1), 32–45. <https://doi.org/10.1080/13607863.2014.909383> |
| MMPR | Wei, W., & Taormina, R. J. (2014). A new multidimensional measure of personal resilience and its use: Chinese nurse resilience, organizational socialization and career success. *Nursing Inquiry*, *21*(4), 346–357. <https://doi.org/10.1111/nin.12067> |
| MTRR-99 | Harvey, M. R., Liang, B., Harney, P. A., Koenen, K., Tummala-Narra, P., & Lebowitz, L. (2003). A multidimensional approach to the assessment of trauma impact, recovery and resiliency: Initial psychometric findings. *Journal of Aggression, Maltreatment & Trauma*, *6*(2), 87–109. <https://doi.org/10.1300/J146v06n02_05> |
| PCQ | Luthans, F., & Youssef-Morgan, C. M. (2017). Psychological capital: An evidence-based positive approach. *Annual Review of Organizational Psychology and Organizational Behavior*, *4*(1), 339–366. <https://doi.org/10.1146/annurev-orgpsych-032516-113324> |
| PCQ-12 | Luthans, F., & Youssef-Morgan, C. M. (2017). Psychological capital: An evidence-based positive approach. *Annual Review of Organizational Psychology and Organizational Behavior*, *4*(1), 339–366. <https://doi.org/10.1146/annurev-orgpsych-032516-113324> |
| PFRS | Harms, C., Pooley, J. A., & Cohen, L. (2017). The protective factors for resilience scale (PFRS): Development of the scale. *Cogent Psychology*, *4*(1), 1400415. <https://doi.org/10.1080/23311908.2017.1400415> |
| PR6-16 | Rossouw, P. J., & Rossouw, J. G. (2016). The predictive 6-factor resilience scale: Neurobiological fundamentals and organizational application. *International Journal of Neuropsychotherapy, 4*(1), 31–45. |
| PR6-50 | Rossouw, P. J., & Rossouw, J. G. (2016). The predictive 6-factor resilience scale: Neurobiological fundamentals and organizational application. *International Journal of Neuropsychotherapy, 4*(1), 31–45. |
| PRS | Resnick, B., Galik, E., Dorsey, S., Scheve, A., & Gutkin, S. (2011). Reliability and validity testing of the physical resilience measure. *The Gerontologist*, *51*(5), 643–652. <https://doi.org/10.1093/geront/gnr016> |
| PTGI | Tedeschi, R. G., & Calhoun, L. G. (1996). The posttraumatic growth inventory: Measuring the positive legacy of trauma. *Journal of Traumatic Stress*, *9*(3), 455–471. <https://doi.org/10.1002/jts.2490090305> |
| PTGI-SF | Cann, A., Calhoun, L. G., Tedeschi, R. G., Taku, K., Vishnevsky, T., Triplett, K. N., & Danhauer, S. C. (2010). A short form of the Posttraumatic Growth Inventory. *Anxiety, Stress & Coping*, *23*(2), 127–137. <https://doi.org/10.1080/10615800903094273> |
| PTGI-X | Tedeschi, R. G., Cann, A., Taku, K., Senol‐Durak, E., & Calhoun, L. G. (2017). The Posttraumatic Growth Inventory: A revision integrating existential and spiritual change. *Journal of Traumatic Stress*, *30*(1), 11–18. <https://doi.org/10.1002/jts.22155> |
| R-MATS | Department of Educational Psychology, Faculty of Education, University of Pretoria, Pretoria, South Africa, & Mampane, M. R. (2014). Factors contributing to the resilience of middle-adolescents in a South African township: Insights from a resilience questionnaire. *South African Journal of Education*, *34*(4), 1–11. <https://doi.org/10.15700/201412052114> |
| RAQ-40 | <https://www.mas-services.org.uk/> |
| RAQ-8 | <https://www.mas-services.org.uk/> |
| RAS | Corrigan, P. W., Giffort, D., Rashid, F., Leary, M., & Okeke, I. (1999). Recovery as a Psychological Construct. *Community Mental Health Journal*, *35*(3), 231–239. <https://doi.org/10.1023/A:1018741302682> |
| RASP | Hurtes, K. P., & Allen, L. R. (2001). Measuring resiliency in youth: The Resiliency Attitudes and Skills Profile. *Therapeutic Recreation Journal, 35*(4), 333–347. |
| RASS | Turner, M., Bowen, P., Ryan, J., & Hayes, P. (2020). Development and validity of a resilience at secondary school scale. *Australian Journal of Education*, *64*(1), 40–53. <https://doi.org/10.1177/0004944119895818> |
| RAU | Turner, M., Holdsworth, S., & Scott-Young, C. M. (2017). Resilience at University: The development and testing of a new measure. *Higher Education Research & Development*, *36*(2), 386–400. <https://doi.org/10.1080/07294360.2016.1185398> |
| RESI-M | Palomar L. J., & Gómez V. N. E. (2010). Desarrollo de una escala de medición de la resiliencia con mexicanos (RESI-M) [Construction of a measurement scale of resilience in Mexicans (RESI-M)]. *Interdisciplinaria 27*, 7–22. |
| RPFC | Powell, K. M., Rahm-Knigge, R. L., & Conner, B. T. (2021). Resilience Protective Factors Checklist (RPFC): Buffering Childhood Adversity and Promoting Positive Outcomes. *Psychological Reports*, *124*(4), 1437–1461. <https://doi.org/10.1177/0033294120950288> |
| RRC-ARM | Liebenberg, L., & Moore, J. C. (2018). A Social Ecological Measure of Resilience for Adults: The RRC-ARM. *Social Indicators Research*, *136*(1), 1–19. <https://doi.org/10.1007/s11205-016-1523-y> |
| RS | Wagnild, G., & Young, H. (1993). Development and psychometric evaluation of the Resilience Scale. *Journal of nursing measurement, 1*(2), 165-178. |
| RS-10 | The Resilience Center. Available from: http://www. resiliencecenter.com/assessments/resilience-scale-forchildren-rs10/. |
| RS-11 | Schumacher, J., Leppert, K., Gunzelmann, T., Strauß, B., & Brähler, E. (2005). Die Resilienzskala - Ein Fragebogen zur Erfassung der psychischen Widerstandsfähigkeit als Personmerkmal [The resilience scale - a questionnaire to assess psychological resilience as a personal characteristic]. *Zeitschrift für Klinische Psychologie, Psychiatrie und Psychotherapie, 53*(1), 16-39. |
| RS-14 | Wagnild, G. (2009). *The Resilience Scale user's guide for the US English version of the Resilience Scale and the 14-item Resilience Scale (RS–14)*. Resilience Center. |
| RS-5 | Von Eisenhart Rothe, A., Zenger, M., Lacruz, M. E., Emeny, R., Baumert, J., Haefner, S., & Ladwig, K.-H. (2013). Validation and development of a shorter version of the resilience scale RS-11: Results from the population-based KORA–age study. *BMC Psychology*, *1*(1), 25. <https://doi.org/10.1186/2050-7283-1-25> |
| RSA (2003) | Friborg, O., Hjemdal, O., Rosenvinge, J. H., & Martinussen, M. (2003). A new rating scale for adult resilience: What are the central protective resources behind healthy adjustment? *International Journal of Methods in Psychiatric Research*, *12*(2), 65–76. <https://doi.org/10.1002/mpr.143> |
| RSA (2005) | Friborg, O., Barlaug, D., Martinussen, M., Rosenvinge, J. H., & Hjemdal, O. (2005). Resilience in relation to personality and intelligence. *International Journal of Methods in Psychiatric Research*, *14*(1), 29–42. <https://doi.org/10.1002/mpr.15> |
| RSAS | Jew, C. L., Green, K. E., & Kroger, J. (1999). Development and Validation of a Measure of Resiliency. *Measurement and Evaluation in Counseling and Development*, *32*(2), 75–89. <https://doi.org/10.1080/07481756.1999.12068973> |
| RSCA | Prince-Embury, S. (2008). The Resiliency Scales for Children and Adolescents, Psychological Symptoms, and Clinical Status in Adolescents. *Canadian Journal of School Psychology*, *23*(1), 41–56. <https://doi.org/10.1177/0829573508316592> |
| RSES | Johnson, D. C., Polusny, M. A., Erbes, C. R., King, D., King, L., Litz, B. T., Schnurr, P. P., Friedman, M., Pietrzak, R. H., & Southwick, S. M. (2011). Development and Initial Validation of the Response to Stressful Experiences Scale. *Military Medicine*, *176*(2), 161–169. <https://doi.org/10.7205/MILMED-D-10-00258> |
| RSS | Maltby, J., Day, L., & Hall, S. (2015). Refining Trait Resilience: Identifying Engineering, Ecological, and Adaptive Facets from Extant Measures of Resilience. *PLOS ONE*, *10*(7), e0131826. <https://doi.org/10.1371/journal.pone.0131826> |
| RSYA | Prince-Embury, S., Saklofske, D. H., & Nordstokke, D. W. (2017). The Resiliency Scale for Young Adults. *Journal of Psychoeducational Assessment*, *35*(3), 276–290. <https://doi.org/10.1177/0734282916641866> |
| SEARS-A SEARS-A-S SEARS-C SEARS-C-S SEARS-P SEARS-P-S SEARS-T SEARS-T-S | Nese, R. N. T., Doerner, E., Romer, N., Kaye, N. C., Merrell, K. W., & Tom, K. M. (2012). Social emotional assets and resilience scales: Development of a strength-based short-form behavior rating scale system. *Journal for Educational Research Online*, *4*(1), 124–139. <https://doi.org/10.25656/01:7054> |
| SPF | Ponce-Garcia, E., Madewell, A. N., & Kennison, S. M. (2015). The Development of the Scale of Protective Factors: Resilience in a Violent Trauma Sample. *Violence and Victims*, *30*(5), 735–755. <https://doi.org/10.1891/0886-6708.VV-D-14-00163> |
| STARS | Lock, S., Rees, C. S., & Heritage, B. (2020). Development and validation of a brief measure of psychological resilience: The state–trait assessment of resilience scale. *Australian Psychologist*, *55*(1), 10–25. <https://doi.org/10.1111/ap.12434> |
| TRAS | Johnson, J., Gooding, P. A., Wood, A. M., & Tarrier, N. (2010). Resilience as positive coping appraisals: Testing the schematic appraisals model of suicide (SAMS). *Behaviour Research and Therapy*, *48*(3), 179–186. <https://doi.org/10.1016/j.brat.2009.10.007> |
| TRS-C | Madsen, M. D., & Abell, N. (2010). Trauma Resilience Scale: Validation of Protective Factors Associated With Adaptation Following Violence. *Research on Social Work Practice*, *20*(2), 223–233. <https://doi.org/10.1177/1049731509347853> |
| WFRQ | Walsh, F. (2016). Applying a Family Resilience Framework in Training, Practice, and Research: Mastering the Art of the Possible. *Family Process*, *55*(4), 616–632. <https://doi.org/10.1111/famp.12260> |
| WRI | McLarnon, M. J. W., & Rothstein, M. G. (2013). Development and Initial Validation of the Workplace Resilience Inventory. *Journal of Personnel Psychology*, *12*(2), 63–73. <https://doi.org/10.1027/1866-5888/a000084> |
