# Supplementary material for: Reverse‐engineering psychological resilience: A review and quantitative evaluation of psychometric instruments used in resilience research
Source: Appl Psychol Health Well Being. 2026 Jul 1;18(4):e70174. doi: 10.1111/aphw.70174 (PMC13321141; doi:10.1111/aphw.70174)
Supplement: Supplementary file 8 — Table S2. Excluded resilience questionnaires, reference and reason for exclusion [file APHW-18-0-s002.docx]

Table S2. Excluded resilience questionnaires, reference and reason for exclusion

| Abbreviation | Reference | Reason for exclusion |
| --- | --- | --- |
| CFSM | Yeung, J.W.K, Lee, S.S.W., Lee, E.M.S., & DeFrain, J. (2012). Development and validation of a Chinese family strengths measure for family services intervention in Hong Kong. *Revista de Cercetare si Interventie Sociala*, 36, 7-30. | Could not be retrieved and no response from the authors |
| ERS | Bromley, E., Johnson, J. G., & Cohen, P. (2006). Personality strengths in adolescence and decreased risk of developing mental health problems in early adulthood. *Comprehensive Psychiatry*, *47*(4), 315–324. <https://doi.org/10.1016/j.comppsych.2005.11.003> | Could not be retrieved and no response from the authors |
| FaRE | Faccio, F., Gandini, S., Renzi, C., Fioretti, C., Crico, C., & Pravettoni, G. (2019). Development and validation of the Family Resilience (FaRE) Questionnaire: An observational study in Italy. *BMJ Open*, *9*(6), 1–9. <https://doi.org/10.1136/bmjopen-2018-024670> | Could not be retrieved and no response from the authors |
| FRQ | Bu T., & Liu H. (2019). Development of family resilience questionnaire. *Psychology, 7*. 173–182. | Was mentioned in the review article by Zhou et al. (2020), but could not be found. |
| HKRQ | Constantine, N., Benard, B., & Diaz, M. (1999). Measuring protective factors and resilience traits in youth: The healthy kids resilience assessment. *In Seventh Annual Meeting of the Society for Prevention Research*, 3–15. | Another version of the CHKS |
| PR | Windle, G., Markland, D. A., & Woods, R. T. (2008). Examination of a theoretical model of psychological resilience in older age. *Aging & Mental Health*, *12*(3), 285–292. <https://doi.org/10.1080/13607860802120763> | Could not be retrieved and no response from the authors |
| READ | Hjemdal, O., Friborg, O., Stiles, T. C., Martinussen, M., & Rosenvinge, J. H. (2006). A new scale for adolescent resilience: Grasping the central protective resources behind healthy development. *Measurement and Evaluation in Counseling and Development*, *39*(2), 84–96. <https://doi.org/10.1080/07481756.2006.11909791> | Could not be retrieved and no response from the authors |
| RIM | Ryan, L., & Caltabiano, M. L. (2009). Development of a new resilience scale: The Resilience in Midlife Scale (RIM Scale). *Asian Social Science*, *5*(11), 39-51. <https://doi.org/10.5539/ass.v5n11p39> | Could not be retrieved and no response from the authors |
| RMP-CC | Ye, Z. J., Qiu, H. Z., Li, P. F., Liang, M. Z., Wang, S. N., & Quan, X. M. (2017). Resilience model for parents of children with cancer in mainland China - An exploratory study. *European Journal of Oncology Nursing*, *27*, 9–16. <https://doi.org/10.1016/j.ejon.2017.01.002> | Could not be retrieved and no response from the authors |
| RYDM | Furlong, M. J., Ritchey, K. M., & O’Brennan, L. M. (2009). Developing norms for the California Resilience Youth Development Module: Internal assets and school resources subscales. *The California School Psychologist*, *14*(1), 35–46. <https://doi.org/10.1007/BF03340949> | Another version of the CHKS |
| YR: ADS | Donnon, T., & Hammond, W. (2007). A psychometric assessment of the self-reported Youth Resiliency: Assessing Developmental Strengths Questionnaire. *Psychological Reports*, *100*(3), 963–978. <https://doi.org/10.2466/pr0.100.3.963-978> | Could not be retrieved and no response from the authors |
