# Supplementary material for: Reverse‐engineering psychological resilience: A review and quantitative evaluation of psychometric instruments used in resilience research
Source: Appl Psychol Health Well Being. 2026 Jul 1;18(4):e70174. doi: 10.1111/aphw.70174 (PMC13321141; doi:10.1111/aphw.70174)
Supplement: Supplementary file 9 — Table S3. Percentage distribution of each category of the framework for each scale for raters 2–5 and items with reached agreed [file APHW-18-0-s005.docx]

Table S3. Percentage distribution of each category of the framework for each scale for raters 2-5 and items with reached agreed

|  | **Process** | | | | | **Personality / Trait** | | | | | | **Environment** | | |  | |
| --- | --- | --- | --- | --- | --- | --- | --- | --- | --- | --- | --- | --- | --- | --- | --- | --- |
| **Scale** | **1** | **2** | **3** | **4** | $\boldsymbol{\Sigma}$ **Proc.** | **5** | **6** | **7** | **8** | **9** | $\boldsymbol{\Sigma}$**Trait** | **10** | **11** | $\boldsymbol{\Sigma}$ **Env.** | **Agreement** | **Disagreement** |
| 5×5RS | 0 | 0 | 0 | 0 | 0 | 24 | 4 | 28 | 0 | 28 | 83.99 | 0 | 0 | 0 | 84 | 16 |
| 7C | 28.57 | 0 | 0 | 0 | 28.57 | 0 | 14.29 | 0 | 14.29 | 0 | 28.57 | 14.29 | 0 | 14.29 | 71.43 | 28.57 |
| ARM-R | 0 | 0 | 0 | 0 | 0 | 5.88 | 0 | 0 | 5.88 | 0 | 11.76 | 41.18 | 5.88 | 47.06 | 58.82 | 41.18 |
| ARQ | 4.55 | 0 | 1.13 | 0 | 5.68 | 0 | 6.82 | 18.18 | 6.82 | 9.09 | 40.92 | 29.55 | 0 | 29.55 | 76.14 | 23.86 |
| ARS | 9.52 | 0 | 4.76 | 0 | 14.28 | 28.57 | 9.52 | 14.29 | 0 | 14.29 | 66.66 | 0 | 0 | 0 | 80.95 | 19.05 |
| ARS-30 | 16.67 | 0 | 0 | 0 | 16.67 | 0 | 10 | 0 | 0 | 0 | 10 | 0 | 0 | 0 | 26.67 | 73.33 |
| BPFI | 20 | 0 | 0 | 0 | 20 | 6.66 | 6.66 | 6.66 | 0 | 0 | 19.99 | 33.33 | 13.33 | 46.66 | 86.67 | 13.33 |
| BRCS | 50 | 0 | 25 | 0 | 75 | 0 | 0 | 0 | 0 | 25 | 25 | 0 | 0 | 0 | 100 | 0 |
| BRS | 100 | 0 | 0 | 0 | 100 | 0 | 0 | 0 | 0 | 0 | 0 | 0 | 0 | 0 | 100 | 0 |
| BURS | 46.67 | 0 | 3.34 | 3.34 | 53.34 | 3.34 | 0 | 0 | 3.34 | 0 | 6.67 | 0 | 0 | 0 | 60 | 40 |
| CD-RISC-10 | 60 | 0 | 10 | 0 | 70 | 0 | 0 | 0 | 0 | 0 | 0 | 0 | 0 | 0 | 70 | 30 |
| CD-RISC-2 | 100 | 0 | 0 | 0 | 100 | 0 | 0 | 0 | 0 | 0 | 0 | 0 | 0 | 0 | 100 | 0 |
| CD-RISC-25 | 36 | 0 | 4 | 0 | 40 | 4 | 4 | 0 | 8 | 4 | 20 | 4 | 0 | 4 | 64 | 36 |
| CHKS | 4.25 | 0 | 0 | 0 | 4.25 | 4.25 | 10.64 | 2.13 | 10.64 | 2.13 | 29.79 | 40.43 | 0 | 40.43 | 74.47 | 25.53 |
| CYRM-R (Child) | 0 | 0 | 0 | 0 | 0 | 0 | 11.77 | 5.88 | 23.53 | 0 | 41.17 | 23.53 | 5.88 | 29.41 | 70.59 | 29.41 |
| CYRM-R (Youth) | 0 | 0 | 0 | 0 | 0 | 5.88 | 5.88 | 0 | 5.88 | 0 | 17.65 | 29.41 | 5.88 | 35.3 | 52.94 | 47.06 |
| DARS | 0 | 0 | 0 | 0 | 0 | 21.74 | 4.35 | 17.39 | 17.39 | 4.35 | 65.21 | 8.69 | 0 | 8.69 | 73.91 | 26.09 |
| DRS-15 | 0 | 0 | 0 | 0 | 0 | 40 | 26.67 | 0 | 0 | 0 | 66.67 | 0 | 0 | 0 | 66.67 | 33.33 |
| DRS-30 | 3.33 | 0 | 0 | 0 | 3.33 | 23.33 | 40 | 0 | 3.33 | 0 | 66.66 | 0 | 0 | 0 | 70 | 30 |
| DRS-45 | 2.22 | 0 | 0 | 0 | 2.22 | 22.22 | 31.11 | 0 | 6.67 | 2.22 | 62.22 | 2.22 | 0 | 2.22 | 66.67 | 33.33 |
| ER | 3.45 | 0 | 0 | 0 | 3.45 | 3.45 | 3.45 | 24.14 | 3.45 | 17.24 | 51.72 | 0 | 0 | 0 | 55.17 | 44.83 |
| ER-11 | 9.09 | 0 | 0 | 0 | 9.09 | 54.55 | 0 | 9.09 | 0 | 0 | 63.64 | 0 | 0 | 0 | 72.73 | 27.27 |
| ER89 | 7.14 | 0 | 0 | 0 | 7.14 | 42.86 | 7.14 | 14.28 | 7.14 | 0 | 71.43 | 0 | 0 | 0 | 78.57 | 21.43 |
| ER89-R | 10 | 0 | 0 | 0 | 10 | 50 | 10 | 10 | 10 | 0 | 80 | 0 | 0 | 0 | 90 | 10 |
| ERESMA | 17.78 | 0 | 2.22 | 0 | 20 | 0 | 4.44 | 0 | 11.11 | 2.22 | 17.78 | 15.56 | 8.89 | 24.45 | 62.22 | 37.78 |
| ERS-15 | 40 | 0 | 0 | 13.34 | 53.34 | 0 | 0 | 0 | 13.34 | 13.34 | 26.67 | 0 | 0 | 0 | 80 | 20 |
| FRA | 13.79 | 0 | 3.45 | 0 | 17.24 | 0 | 0 | 3.45 | 3.45 | 0 | 6.9 | 24.14 | 3.45 | 27.59 | 51.72 | 48.28 |
| FRAS | 20.89 | 0 | 0 | 0 | 20.89 | 5.97 | 0 | 1.49 | 10.45 | 1.49 | 19.4 | 23.88 | 4.48 | 28.36 | 68.66 | 31.34 |
| FRI | 0 | 0 | 5 | 0 | 5 | 0 | 5 | 10 | 15 | 5 | 35 | 25 | 0 | 25 | 65 | 35 |
| FRS-V | 33.33 | 0 | 0 | 0 | 33.33 | 0 | 0 | 0 | 0 | 0 | 0 | 50 | 0 | 50 | 83.33 | 16.67 |
| FRS16 | 31.25 | 0 | 0 | 0 | 31.25 | 6.25 | 0 | 0 | 0 | 0 | 6.25 | 25 | 6.25 | 31.25 | 68.75 | 31.25 |
| HCRS | 18.75 | 0 | 12.5 | 0 | 31.25 | 6.25 | 18.75 | 0 | 0 | 0 | 25 | 25 | 0 | 25 | 81.25 | 18.75 |
| HGRS | 44.44 | 0 | 44.44 | 0 | 88.88 | 0 | 0 | 0 | 0 | 0 | 0 | 0 | 11.11 | 11.11 | 100 | 0 |
| IFCR | 12 | 0 | 0 | 0 | 12 | 1.33 | 8 | 6.66 | 0 | 16 | 31.99 | 8 | 13.33 | 21.33 | 65.33 | 34.67 |
| MeRS | 10.81 | 0 | 2.7 | 0 | 13.51 | 5.41 | 18.92 | 0 | 2.7 | 2.7 | 29.73 | 8.11 | 0 | 8.11 | 51.35 | 48.65 |
| MIIRM | 22.73 | 0 | 0 | 0 | 22.73 | 0 | 0 | 9.09 | 13.64 | 0 | 22.73 | 27.27 | 9.09 | 36.36 | 81.82 | 18.18 |
| MMPR | 27.5 | 0 | 2.5 | 10 | 40 | 15 | 20 | 0 | 0 | 0 | 35 | 0 | 0 | 0 | 75 | 25 |
| MTRR-99 | 12.12 | 0 | 0 | 0 | 12.12 | 0 | 3.03 | 3.03 | 10.1 | 11.11 | 27.27 | 0 | 1.01 | 1.01 | 40.4 | 59.6 |
| PCQ | 20.83 | 0 | 0 | 4.17 | 25 | 0 | 8.33 | 16.67 | 0 | 4.17 | 29.16 | 0 | 0 | 0 | 54.17 | 45.83 |
| PCQ-12 | 8.33 | 0 | 0 | 0 | 8.33 | 0 | 8.33 | 25 | 0 | 8.33 | 41.67 | 0 | 0 | 0 | 50 | 50 |
| PFRS | 5 | 0 | 0 | 0 | 5 | 0 | 20 | 5 | 5 | 0 | 30 | 30 | 0 | 30 | 65 | 35 |
| PR6-16 | 0 | 0 | 0 | 0 | 0 | 6.25 | 18.75 | 6.25 | 0 | 6.25 | 37.5 | 0 | 12.5 | 12.5 | 50 | 50 |
| PR6-50 | 8 | 0 | 0 | 0 | 8 | 8 | 24 | 8 | 0 | 8 | 47.99 | 2 | 4 | 6 | 62 | 38 |
| PRS | 13.33 | 0 | 20 | 0 | 33.33 | 6.67 | 0 | 0 | 0 | 0 | 6.67 | 6.67 | 0 | 6.67 | 46.67 | 53.33 |
| PTGI | 0 | 0 | 95.24 | 0 | 95.24 | 4.76 | 0 | 0 | 0 | 0 | 4.76 | 0 | 0 | 0 | 100 | 0 |
| PTGI-SF | 0 | 0 | 90 | 0 | 90 | 10 | 0 | 0 | 0 | 0 | 10 | 0 | 0 | 0 | 100 | 0 |
| PTGI-X | 0 | 0 | 96 | 0 | 96 | 4 | 0 | 0 | 0 | 0 | 4 | 0 | 0 | 0 | 100 | 0 |
| R-MATS | 12.5 | 0 | 0 | 0 | 12.5 | 0 | 12.5 | 4.17 | 0 | 8.33 | 25 | 37.5 | 0 | 37.5 | 75 | 25 |
| RAQ-40 | 2.5 | 0 | 0 | 5 | 7.5 | 15 | 25 | 12.5 | 12.5 | 5 | 69.99 | 2.5 | 0 | 2.5 | 80 | 20 |
| RAQ-8 | 0 | 0 | 0 | 0 | 0 | 25 | 37.5 | 12.5 | 0 | 0 | 75 | 12.5 | 0 | 12.5 | 87.5 | 12.5 |
| RAS | 12.5 | 0 | 0 | 4.16 | 16.67 | 4.16 | 20.83 | 8.33 | 0 | 8.33 | 41.66 | 12.5 | 0 | 12.5 | 70.83 | 29.17 |
| RASP | 8.83 | 0 | 0 | 0 | 8.83 | 11.77 | 14.71 | 5.88 | 8.83 | 5.88 | 47.06 | 5.88 | 0 | 5.88 | 61.76 | 38.24 |
| RASS | 15.79 | 0 | 0 | 0 | 15.79 | 0 | 10.53 | 0 | 0 | 10.53 | 21.05 | 10.53 | 0 | 10.53 | 47.37 | 52.63 |
| RAU | 10.53 | 0 | 0 | 0 | 10.53 | 0 | 15.79 | 0 | 5.26 | 0 | 21.05 | 10.53 | 0 | 10.53 | 42.11 | 57.89 |
| RESI-M | 11.63 | 0 | 2.32 | 0 | 13.96 | 4.65 | 20.93 | 18.61 | 2.32 | 2.32 | 48.83 | 16.28 | 0 | 16.28 | 79.07 | 20.93 |
| RPFC | 12 | 0 | 4 | 0 | 16.01 | 0 | 0 | 4 | 0 | 4 | 8.01 | 48 | 0 | 48 | 72 | 28 |
| RRC-ARM | 3.57 | 0 | 0 | 0 | 3.57 | 0 | 14.28 | 7.14 | 3.57 | 0 | 25 | 32.14 | 3.57 | 35.72 | 64.29 | 35.71 |
| RS | 16 | 0 | 0 | 4 | 20 | 12 | 20 | 8 | 4 | 4 | 47.99 | 0 | 0 | 0 | 68 | 32 |
| RS-10 | 0 | 0 | 0 | 10 | 10 | 20 | 30 | 0 | 0 | 20 | 70 | 0 | 0 | 0 | 80 | 20 |
| RS-11 | 9.09 | 0 | 0 | 9.09 | 18.18 | 27.27 | 27.27 | 9.09 | 0 | 9.09 | 72.73 | 0 | 0 | 0 | 90.91 | 9.09 |
| RS-14 | 28.57 | 0 | 0 | 7.14 | 35.71 | 7.14 | 14.29 | 0 | 7.14 | 7.14 | 35.71 | 0 | 0 | 0 | 71.43 | 28.57 |
| RS-5 | 0 | 0 | 0 | 0 | 0 | 60 | 20 | 0 | 0 | 0 | 80 | 0 | 0 | 0 | 80 | 20 |
| RSA (2003) | 11.11 | 0 | 0 | 0 | 11.11 | 0 | 16.67 | 25 | 5.56 | 2.78 | 50 | 19.45 | 0 | 19.45 | 80.56 | 19.44 |
| RSA (2005) | 3.03 | 0 | 0 | 0 | 3.03 | 0 | 15.15 | 24.24 | 0 | 6.06 | 45.45 | 27.27 | 0 | 27.27 | 75.76 | 24.24 |
| RSAS | 8.89 | 2.22 | 2.22 | 0 | 13.33 | 2.22 | 2.22 | 13.33 | 26.67 | 2.22 | 46.67 | 2.22 | 0 | 2.22 | 62.22 | 37.78 |
| RSCA | 7.81 | 0 | 0 | 0 | 7.81 | 1.56 | 1.56 | 9.38 | 12.5 | 3.13 | 28.12 | 10.94 | 0 | 10.94 | 46.88 | 53.12 |
| RSES | 45.46 | 0 | 18.18 | 0 | 63.64 | 0 | 0 | 0 | 0 | 0 | 0 | 0 | 0 | 0 | 63.64 | 36.36 |
| RSS | 33.33 | 0 | 0 | 0 | 33.33 | 33.33 | 16.67 | 0 | 0 | 0 | 50 | 0 | 0 | 0 | 83.33 | 16.67 |
| RSYA | 14 | 0 | 2 | 0 | 16 | 4 | 0 | 14 | 10 | 4 | 32 | 8 | 0 | 8 | 56 | 44 |
| SEARS-A | 14.29 | 0 | 0 | 0 | 14.29 | 0 | 17.15 | 17.15 | 37.15 | 8.57 | 80.01 | 2.86 | 0 | 2.86 | 97.14 | 2.86 |
| SEARS-A-S | 16.67 | 0 | 0 | 0 | 16.67 | 0 | 25 | 25 | 25 | 8.33 | 83.33 | 0 | 0 | 0 | 100 | 0 |
| SEARS-C | 17.14 | 0 | 0 | 0 | 17.14 | 0 | 17.14 | 14.28 | 31.43 | 14.28 | 77.14 | 0 | 0 | 0 | 94.29 | 5.71 |
| SEARS-C-S | 25 | 0 | 0 | 0 | 25 | 0 | 16.67 | 0 | 25 | 33.33 | 75 | 0 | 0 | 0 | 100 | 0 |
| SEARS-P | 15.38 | 0 | 0 | 0 | 15.38 | 0 | 17.95 | 15.38 | 38.46 | 12.82 | 84.61 | 0 | 0 | 0 | 100 | 0 |
| SEARS-P-S | 25 | 0 | 0 | 0 | 25 | 0 | 16.67 | 8.33 | 41.67 | 8.33 | 75 | 0 | 0 | 0 | 100 | 0 |
| SEARS-T | 7.31 | 0 | 0 | 0 | 7.31 | 0 | 19.51 | 21.95 | 34.15 | 12.19 | 87.81 | 0 | 0 | 0 | 95.12 | 4.88 |
| SEARS-T-S | 8.33 | 0 | 0 | 0 | 8.33 | 0 | 16.67 | 25 | 33.33 | 16.67 | 91.67 | 0 | 0 | 0 | 100 | 0 |
| SPF | 0 | 0 | 0 | 0 | 0 | 0 | 45.83 | 25 | 4.16 | 0 | 75 | 12.5 | 0 | 12.5 | 87.5 | 12.5 |
| STARS | 76.92 | 0 | 0 | 7.69 | 84.61 | 0 | 0 | 0 | 0 | 15.38 | 15.38 | 0 | 0 | 0 | 100 | 0 |
| TRAS | 25 | 0 | 0 | 0 | 25 | 0 | 0 | 0 | 0 | 25 | 25 | 33.33 | 0 | 33.33 | 83.33 | 16.67 |
| TRS-C | 12.5 | 0 | 1.56 | 0 | 14.06 | 3.12 | 0 | 4.69 | 3.12 | 7.81 | 18.74 | 35.94 | 10.94 | 46.88 | 79.69 | 20.31 |
| WFRQ | 25 | 0 | 6.25 | 0 | 31.25 | 0 | 3.13 | 0 | 0 | 0 | 3.13 | 31.25 | 6.25 | 37.5 | 71.88 | 28.12 |
| WRI | 15 | 0 | 0 | 0 | 15 | 13.33 | 21.66 | 0 | 0 | 13.33 | 48.32 | 8.33 | 0 | 8.33 | 71.67 | 28.33 |

*Note*. Resilience (1), vulnerability (2), posttraumatic growth (3), resistance (4), openness (5), conscientiousness (6), extraversion (7), agreeableness (8), neuroticism (9), social factors (10), other, non-social factors (11).
